# Mesenchymal progenitors in osteopenias of diverse pathologies: differential characteristics in the common shift from osteoblastogenesis to adipogenesis

**DOI:** 10.1038/srep30186

**Published:** 2016-07-22

**Authors:** Bingdong Sui, Chenghu Hu, Li Liao, Yichen Chen, Xinyi Zhang, Xin Fu, Chenxi Zheng, Meng Li, Ling Wu, Xinyi Zhao, Yan Jin

**Affiliations:** 1 State Key Laboratory of Military Stomatology, Center for Tissue Engineering, Fourth Military Medical University, Xi’an 710032, Shaanxi, China; 2 Xi’an Institute of Tissue Engineering and Regenerative Medicine, Xi’an 710032, Shaanxi, China; 3 Department of Dental Materials, School of Stomatology, Fourth Military Medical University, Xi’an 710032, Shaanxi, China; 4 State Key Laboratory of Military Stomatology, Department of Dental Materials, Fourth Military Medical University, Xi’an 710032, Shaanxi, China

## Abstract

Osteoporosis is caused by pathologic factors such as aging, hormone deficiency or excess, inflammation, and systemic diseases like diabetes. Bone marrow stromal cells (BMSCs), the mesenchymal progenitors for both osteoblasts and adipocytes, are modulated by niche signals. In differential pathologic states, the pathological characteristics of BMSCs to osteoporoses and functional differences are unknown. Here, we detected that trabecular bone loss co-existed with increased marrow adiposity in 6 osteoporotic models, respectively induced by natural aging, accelerated senescence (SAMP6), ovariectomy (OVX), type 1 diabetes (T1D), excessive glucocorticoids (GIOP) and orchidectomy (ORX). Of the *ex vivo* characteristics of BMSCs, the colony-forming efficiency and the proliferation rate in aging, SAMP6, OVX, GIOP and ORX models decreased. The apoptosis and cellular senescence increased except in T1D, with up-regulation of *p53* and *p16* expression. The osteogenesis declined except in GIOP, with corresponding down-regulation of *Runt-related transcription factor 2 (RUNX2)* expression. The adipogenesis increased in 6 osteoporotic models, with corresponding up-regulation of *Peroxisome proliferator activated receptor gamma (PPARγ)* expression. These findings revealed differential characteristics of BMSCs in a common shift from osteoblastogenesis to adipogenesis among different osteoporoses and between sexes, and provide theoretical basis for the functional modulation of resident BMSCs in the regenerative therapy for osteoporosis.

Osteoporosis is caused by various pathologic factors, such as aging^1^,^2^, hormone deficiency or excess^3^,^4^,^5^, inflammation^4^,^6^, and systemic diseases like diabetes^7^,^8^. It is characterized by bone loss with increased marrow adiposity^9^,^10^,^11^. However, this interrelationship depends on certain factors like menopause and diabetes, and demonstrates potential differences among different types of osteoporoses and between sexes^11^,^12^,^13^,^14^. Bone marrow stromal cells (BMSCs) are typically recognized as heterogeneous mesenchymal progenitors for both osteoblasts and marrow adipocytes^15^,^16^. Physiologically, recent studies have identified and fate-mapped specific BMSC subpopulations *in vivo*, demonstrating their function in osteoblastogenesis and adipogenesis in the adult bone marrow^17^,^18^,^19^. Furthermore, in pathological states, studies have shown that dysfunction of BMSCs in colony-forming potential, proliferation, apoptosis, senescence and/or differentiation direction may participate in the development of osteoporosis, and these characteristics are synergistically modulated by local and systemic pathologic signals^1^,^6^,^20^,^21^,^22^. In osteoporoses of diverse pathologies, the pathological characteristics and potential functional differences of BMSCs are unknown.

Aging is notable for an acceleration of bone loss and an accumulation of marrow adipocytes that is attributed to complicated pathology^1^,^2^. It has been well documented that the age-related functional decline of BMSCs includes impaired proliferation and osteogenic potential with a misdirected differentiation toward adipocytes^1^,^21^,^23^. It has also been reported in the stem cell antigen (Sca1)^−/−^ mice that the age-related osteoporosis could be caused by depletion of the BMSC pool^24^. The phenotypes of a murine model of senile osteoporosis, the senescence-accelerated mice-prone 6 (SAMP6) with a AKR/J background, closely replicate the clinical manifestations in human^25^. Compared to the senescence-accelerated mice-resistant 1 (SAMR1), SAMP6 mice demonstrate impairments in osteoblastogenesis and increases in adipogenesis^26^,^27^. Postmenopausal osteoporosis is the most prevalent form of primary osteoporoses^28^. The proliferation and osteogenic potential of BMSCs are reduced with an increased adipogenic potential, which are attributed to the lack of estrogen and the onset of systemic inflammation, as shown in mice after ovariectomy (OVX)^6^,^29^,^30^. Diabetes is a high-incidence metabolic disease with skeletal complications. In particular, type 1 diabetes (T1D) develops bone loss with increased marrow adiposity^7^,^31^,^32^. Notably, some studies have reported differential marrow adipose tissue (MAT) formation in postmenopausal women with diabetes, but clinical findings are few and inconsistent^12^,^14^,^33^. In the streptozotocin (STZ)-induced murine T1D model, reduced proliferation and osteogenic differentiation of BMSCs has been reported, while changes of the adipogenic potential are unknown^20^,^34^. Glucocorticoid-induced osteoporosis (GIOP) is the most common secondary osteoporoses, of which the key feature is the rapid reduction of bone formation and subsequent aggregation of MAT^3^,^35^. Although dexamethasone (DEX) is widely used in inducing both osteogenic and adipogenic differentiation of BMSCs *in vitro*^3^,^36^,^37^, DEX shifts BMSC differentiation from osteoblasts to adipocytes and inhibits BMSC proliferation in GIOP^22^,^38^. Osteoporosis in men is also characterized by bone loss and increased MAT. Compared to postmenopausal women, the skeletal phenotypes of osteoporotic men exhibit sexual differences^5^,^11^,^13^. As far as we know, the changes of BMSC characteristics in both patients and animal models of orchidectomy (ORX) are still unknown.

In this study, we aimed to investigate, in osteoporoses of diverse pathologies, the potential differential interrelationships between bone and MAT and the underlying contributions of BMSCs. We expected to uncover general changes in their characteristics, and putative differences in response to differential pathologic factors and between sexes. We established 6 different types of osteopenias in mice, respectively induced by natural aging, accelerated senescence, OVX, T1D, GIOP and ORX. The bone mass, the marrow adiposity and the bone formation parameters were evaluated. We examined characteristics of the colony-forming efficiency (CFE), the proliferation rate, the apoptosis, the cellular senescence, the osteogenic potential and the adipogenic potential of BMSCs *ex vivo*. Our results indicated dysfunction of BMSCs in the common shift from osteoblastogenesis to adipogenesis in osteoporoses of diverse pathologies, but functional differences existed among different models, particularly in T1D and GIOP.

## Results

### Trabecular bone mass and marrow adiposity in osteopenias with diverse pathologies

At the beginning, we established 6 murine osteoporotic models with diverse pathologies according to previous studies: (1) the natural aging model and (2) the senescence-accelerated SAMP6 model for senile osteoporoses^1^,^39^; (3) the OVX model for postmenopausal osteoporosis^6^; (4) the T1D model and (5) the GIOP model for secondary osteoporoses, of which T1D was induced by multiple low-dose of STZ^40^,^41^; and (6) the ORX model for androgen deficiency-induced osteoporosis^42^. After 4 weeks, the modeled mice developed trabecular bone loss compared to the control groups, as shown by micro-CT images and the corresponding parameters (Supplementary Fig. S1). Notably, the modeled mice suffered from bone loss to different extents, particularly the natural aging mice seem to develop the most severe osteopenia, indicating intergroup differences existed in the 6 types of osteoporoses (Supplementary Fig. S1). Meanwhile, similar bone phenotypes were detected among the control mice, despite the different background between C57 and SAMR1^25^. Hereafter the controls were pooled to allow intergroup comparisons among the modeled mice. Controls were individually shown in supplementary Figs S1–S3, S6–S7 and S9–S11.

**Figure 1:**
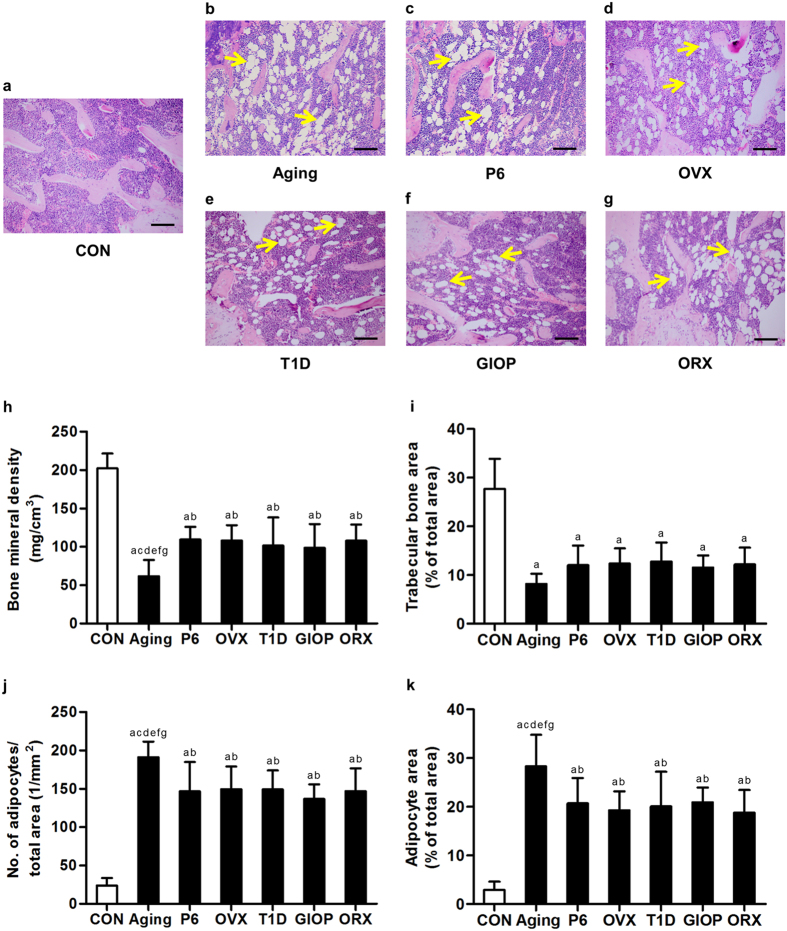
Trabecular bone mass and marrow adiposity. (**a–g**) Representative H&E images demonstrating trabecular bone and marrow adipose tissue in the distal femora of mice. Mice were modeled 4 weeks for the control groups (**a**) and osteoporoses induced by natural aging (**b**), accelerated senescence (SAMP6) (**c**), ovariectomy (OVX) (**d**), type 1 diabetes (T1D) (**e**), excessive glucocorticoids (GIOP) (**f**), and orchidectomy (ORX) (**g**). The unstained area in the bone marrow represented empty spaces occupied by adipocytes with yellow allows indicating. Bars: 100 μm. (**h**) Analyzed by micro-CT, the parameter of bone mineral density (BMD) in the distal metaphysis of femora demonstrating bone loss in all of the 6 types of osteoporoses, among which natural aging mice exhibited the most severe bone loss. (**i–k**) In bone histomorphometric analysis by H&E staining, the corresponding parameters of trabecular bone area over total area (**i**), number of adipocytes over total area (**j**) and adipocyte area over total area (**k**) showing osteoporotic bone loss with increased marrow adiposity in 6 different types of osteoporoses, among which natural aging mice developed the most adipocytes. Data represents mean ± standard deviation (SD). n = 6 per group. ^a^*P* < 0.05 with the control groups; ^b^*P* < 0.05 with the natural aging group; ^c^*P* < 0.05 with the SAMP6 group; ^d^*P* < 0.05 with the OVX group; ^e^*P* < 0.05 with the T1D group; ^f^*P* < 0.05 with the GIOP group; ^g^*P* < 0.05 with the ORX group.

We confirmed the interrelationship between bone loss and increased MAT in these 6 different types of osteopenias in bone histomorphometric analysis (Fig. 1a–g). Quantitative analysis showed a significant reduction of trabecular bone mineral density (BMD) by micro-CT (Fig. 1h) and a reduced percentage of trabecular bone area by H&E staining (Fig. 1i) in each model compared to the control group. Notably, natural aging mice indeed developed the most severe osteopenia, and mice of other models developed similar bone loss. Evaluations on marrow adiposity revealed increases of adipocyte number and area in all of the 6 osteoporotic models, with natural aging mice developing the most MAT (Fig. 1j,k). No sexual differences were detected between OVX and ORX models. The bone mass and marrow adiposity were comparable in the control groups of the 6 osteoporotic models (Supplementary Fig. S2).

### Bone formation parameters in osteopenias with diverse pathologies

**Figure 2:**
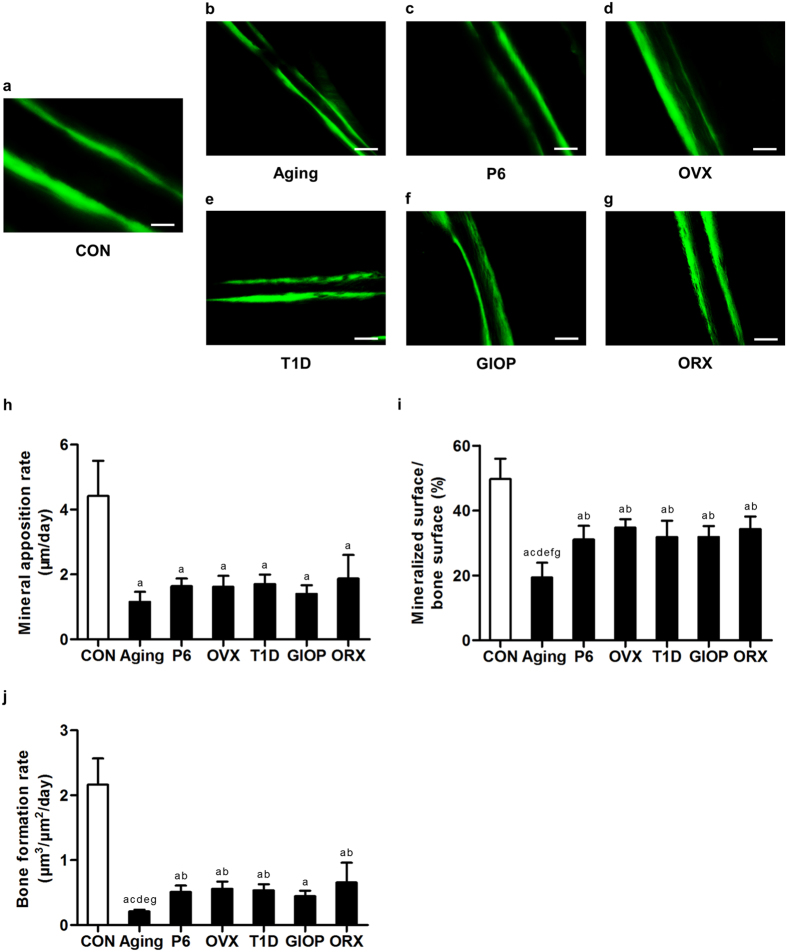
Cortical bone formation parameters. (**a–g**) Representative calcein labeling images demonstrating cortical bone formation in the cortical endosteum of the distal femora of mice. Mice were modeled 4 weeks for the control groups (**a**) and osteoporoses induced by natural aging (**b**), accelerated senescence (SAMP6) (**c**), ovariectomy (OVX) (**d**), type 1 diabetes (T1D) (**e**), excessive glucocorticoids (GIOP) (**f**), and orchidectomy (ORX). (**g**) Mice accepted double intraperitoneal injection of 20 mg/kg calcein at 16 days and 2 days prior to sacrifice. Calcein was dissolved in PBS at 2 mg/ml supplemented with 1 mg/ml NaHCO_3_. Bars: 100 μm. (**h–j**) The corresponding parameters of mineral apposition rate (**h**), mineralized surface over bone surface (**i**) and bone formation rate (**j**) showing impaired cortical bone formation in 6 different types of osteoporoses. The cortical bone formation of natural aging mice declined the most. Data represents mean ± standard deviation (SD). n = 6 per group. ^a^*P* < 0.05 with the control groups; ^b^*P* < 0.05 with the natural aging group; ^c^*P* < 0.05 with the SAMP6 group; ^d^*P* < 0.05 with the OVX group; ^e^*P* < 0.05 with the T1D group; ^f^*P* < 0.05 with the GIOP group; ^g^*P* < 0.05 with the ORX group.

We next investigated whether the increased MAT was accompanied by impaired osteoblastogenesis in diverse osteopenias. Analyses by calcein labeling demonstrated reduced bone formation in the endosteum of the cortical bone in the 6 models (Fig. 2a–g), as shown by the corresponding parameters of mineral apposition rate (MAR) (Fig. 2h), mineralized surface over bone surface (MS/BS) (Fig. 2i) and bone formation rate (BFR) (Fig. 2j). Moreover, MS/BS and BFR parameters in natural aging mice were significantly lower compared to most of other models, indicating that the cortical bone formation declined the most in the natural aging model. No sexual differences were detected between OVX and ORX models. The cortical bone formation was paralleled in the control groups of the 6 osteoporotic models (Supplementary Fig. S3). Similar results were detected regarding the trabecular bone formation velocity (Supplementary Fig. S4). These results suggested a common shift from osteoblastogenesis to adipogenesis in osteopenias of diverse pathologies.

### Colony forming efficiency of BMSCs derived from different models

**Figure 3:**
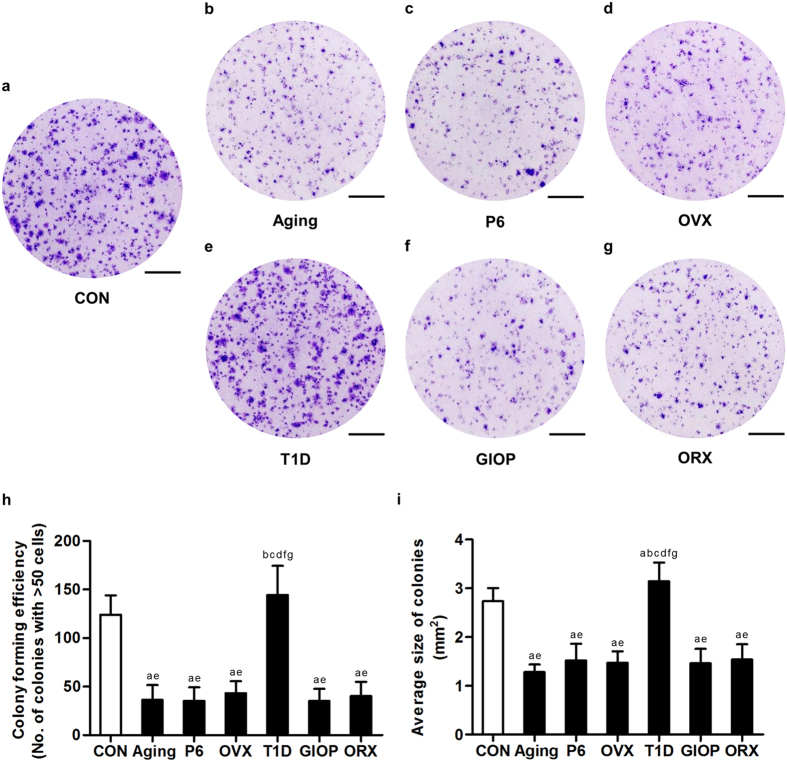
Colony-forming efficiency of bone marrow stromal cells (BMSCs). (**a–g**) Representative images demonstrating colonies formed by primary murine BMSCs derived from the control groups (**a**) and osteoporoses induced by natural aging (**b**), accelerated senescence (SAMP6) (**c**), ovariectomy (OVX) (**d**), type 1 diabetes (T1D) (**e**), excessive glucocorticoids (GIOP) (**f**), and orchidectomy (ORX) (**g**). Mice were sacrificed after 4 weeks of modeling. Primary bone marrow cells were seeded at 1 × 10^5^ cells/cm^2^ after lysis of red blood cells. Bars: 1 cm. (**h,i**) The corresponding parameters of number of colonies (**h**) and average size of colonies (**i**) showing reduced colony-forming efficiency of BMSCs in osteoporoses except in T1D. Colonies with over 50 cells were taken into consideration. Data represents mean ± standard deviation (SD). n = 6 per group. ^a^*P* < 0.05 with the control groups; ^b^*P* < 0.05 with the natural aging group; ^c^*P* < 0.05 with the SAMP6 group; ^d^*P* < 0.05 with the OVX group; ^e^*P* < 0.05 with the T1D group; ^f^*P* < 0.05 with the GIOP group; ^g^*P* < 0.05 with the ORX group.

To examine the underlying contributions and functional differences of BMSCs in diverse osteoporoses, we firstly isolated and identified BMSCs with surface markers. Analyzed by flow cytometry, BMSCs expressed surface makers representing mesenchymal stem cells, including CD29, CD106 and stem cell antigen-1 (SCA-1), but were negative for hematopoietic markers CD11b, CD34 and CD45 (Supplementary Fig. S5). We then compared the CFE of BMSCs derived from the 6 different osteoporotic models (Fig. 3a–g). We found that except those from T1D mice, BMSCs exhibited reduced number (Fig. 3h) and size (Fig. 3i) of the colonies formed, indicating the ability to grow in a density independent manner was inhibited. BMSCs from natural aging mice showed comparable efficiency of colony formation to those derived from OVX, ORX, SAMP6 and GIOP models, but BMSCs from T1D mice formed larger colonies even compared to the control group (Fig. 3i). The CFE was paralleled in the control groups of the 6 osteoporotic models (Supplementary Fig. S6).

### Proliferation and apoptosis of BMSCs derived from different models

**Figure 4:**
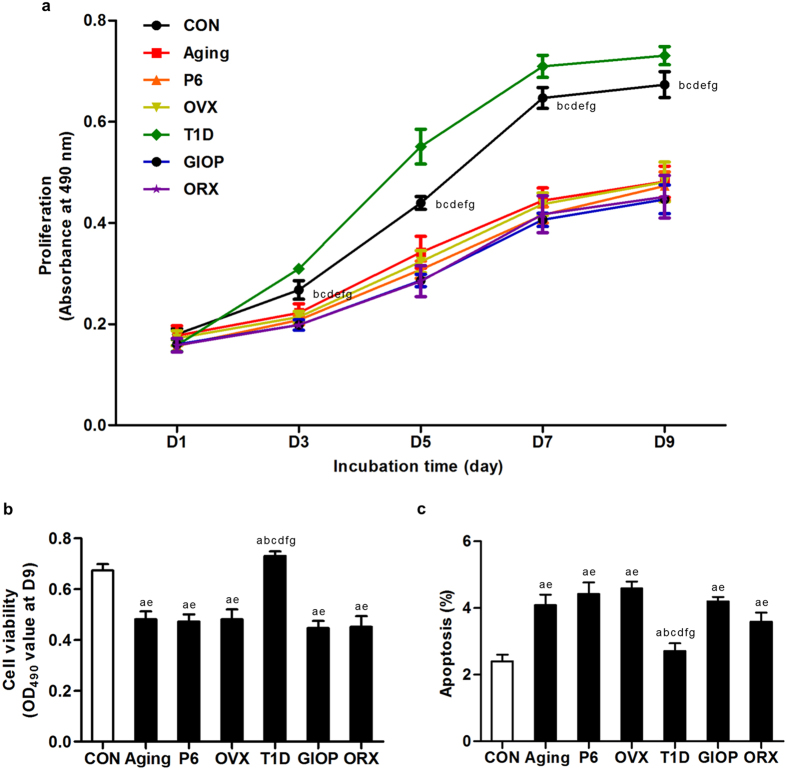
Proliferation rate of bone marrow stromal cells (BMSCs). (**a**) Representative MTT curves demonstrating proliferation of murine BMSCs derived from the control groups and osteoporoses induced by natural aging, accelerated senescence (SAMP6), ovariectomy (OVX), type 1 diabetes (T1D), excessive glucocorticoids (GIOP), and orchidectomy (ORX). Mice were sacrificed after 4 weeks of modeling. 1^st^-passaged BMSCs were seeded at 2 × 10^3^ cells/well in 96-well plates and the cell viability was evaluated at the same time points in Day-1, 3, 5, 7, and 9. Statistical analysis was performed between the control group and each of the osteoporotic groups. (**b**) The values of optical density at 490 nm in the MTT test at Day-9 showing decreased proliferation rate of BMSCs in osteoporoses except in T1D. Intergroup comparisons among the osteoporotic groups were demonstrated. (**c**) Flow cytometry analysis showed increased apoptosis of BMSCs in osteoporoses except in T1D. 1^st^ passaged BMSCs were harvested 3 days after seeding and evaluated by FITC-conjugated Annexin V and propidium iodide (PI) double staining, and the percentages of early apoptotic (FITC^+^PI^−^) plus late apoptotic/necrotic (FITC^+^PI^+^) cells were expressed as apoptotic BMSC percentages. Data represents mean ± standard deviation (SD). n = 6 per group. ^a^*P* < 0.05 with the control groups; ^b^*P* < 0.05 with the natural aging group; ^c^*P* < 0.05 with the SAMP6 group; ^d^*P* < 0.05 with the OVX group; ^e^*P* < 0.05 with the T1D group; ^f^*P* < 0.05 with the GIOP group; ^g^*P* < 0.05 with the ORX group.

We then examined the proliferation rate of BMSCs by depicting the methyl thiazolyl tetrazolium (MTT) curves. Compared to the control group, the proliferation rates of BMSCs derived from osteoporotic groups decreased, except those from T1D mice (Fig. 4a). Intergroup analysis on cell viability at Day-9 (Fig. 4b) among the osteoporotic models demonstrated similar decline of proliferation of BMSCs, except in T1D. Further analysis by flow cytometry showed increased apoptosis of BMSCs derived from all osteoporotic models except T1D (Fig. 4c). No difference was detected among the control groups in the proliferation rate and apoptosis (Supplementary Figs S7 and S8).

### Cellular senescence of BMSCs derived from different models

**Figure 5:**
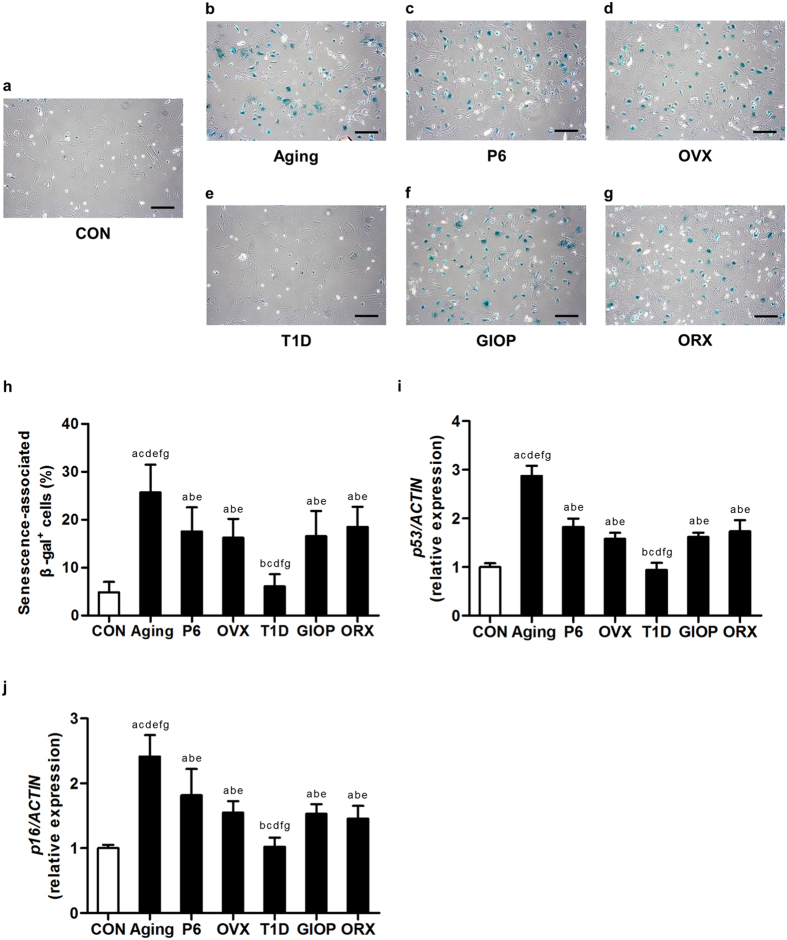
Cellular senescence of bone marrow stromal cells (BMSCs). (**a–g**) Representative beta-galactosidase (β-gal) staining images demonstrating cellular senescence of murine BMSCs derived from the control groups (**a**) and osteoporoses induced by natural aging (**b**), accelerated senescence (SAMP6) (**c**), ovariectomy (OVX) (**d**), type 1 diabetes (T1D) (**e**), excessive glucocorticoids (GIOP) (**f**), and orchidectomy (ORX) (**g**). Mice were sacrificed after 4 weeks of modeling. 1^st^-passaged BMSCs were seeded at 1 × 10^5^ cells/well in 12-well plates and were stained for β-gal. The senescent cells were positively stained (green). Bars: 200 μm. (**h**) The corresponding percentage of senescence-associated β-gal positive cells over total cells showing increased senescence of BMSCs in osteoporoses except in T1D. BMSCs from natural aging mice developed the highest senescent percentage. (**i,j**) Quantitative real-time polymerase chain reaction (qRT-PCR) analysis of the mRNA expression level of *p53* (**i**) and *p16* (**j**). The corresponding values showing increased senescence of BMSCs in osteoporoses except in T1D. BMSCs from natural aging mice expressed the highest levels of *p53* and *p16*. The expressions of *p53* and *p16* were normalized to that of *ACTIN*. Data represents mean ± standard deviation (SD). n = 6 per group. ^a^*P* < 0.05 with the control groups; ^b^*P* < 0.05 with the natural aging group; ^c^*P* < 0.05 with the SAMP6 group; ^d^*P* < 0.05 with the OVX group; ^e^*P* < 0.05 with the T1D group; ^f^*P* < 0.05 with the GIOP group; ^g^*P* < 0.05 with the ORX group.

Next, we evaluated the cellular senescence of BMSCs by the senescence-associated beta-galactosidase (SA-β-gal) analysis. As depicted by β-gal staining, BMSCs from the models of OVX, ORX, natural aging, SAMP6 and GIOP demonstrated higher percentages of senescent cells, but the senescent state of BMSCs from T1D mice was comparable with those of control (Fig. 5a–g). Quantitative analysis on the percentage of SA-β-gal positive cells over total cells revealed the most senescent cells in BMSCs from natural aging mice (Fig. 5h). Quantitative real-time polymerase chain reaction (qRT-PCR) analysis on the senescent marker genes *p53* and *p16* indicated up-regulation in cellular senescence of BMSCs, except those from T1D mice. Correspondingly, we also detected the highest mRNA expression levels of *p53* and *p16* in BMSCs derived from natural aging mice (Fig. 5i, j). No difference was detected between OVX and ORX models and among the control groups (Supplementary Fig. S9).

### Osteogenic differentiation of BMSCs derived from different models

**Figure 6:**
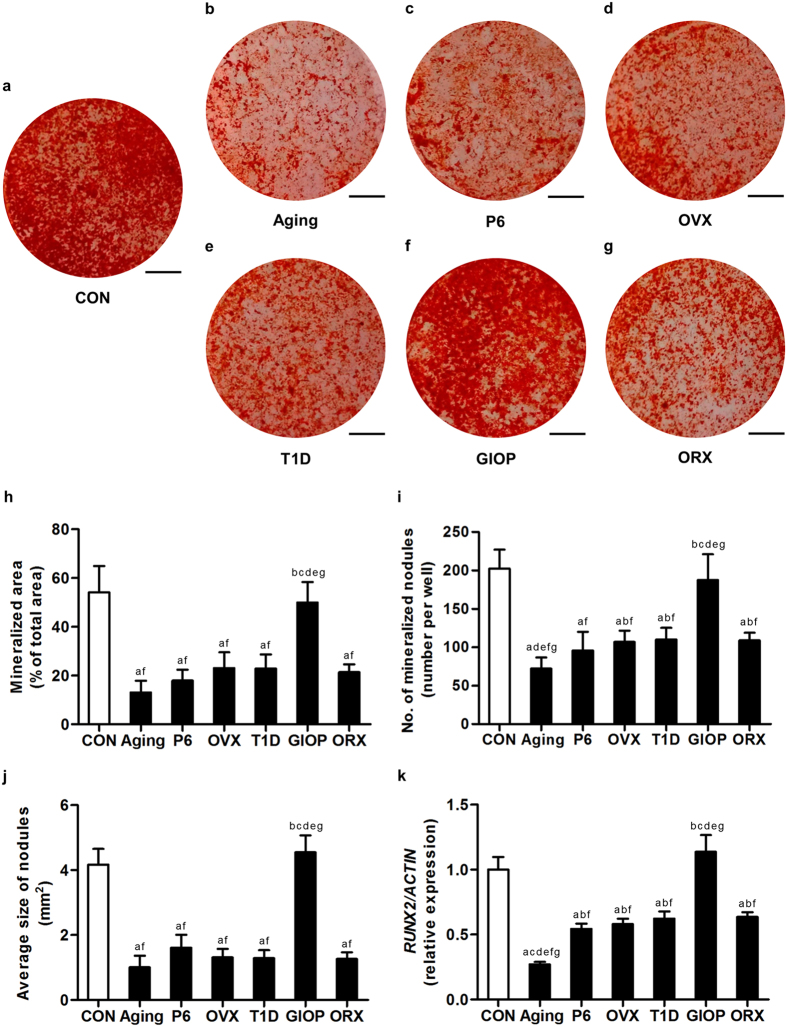
Osteogenic differentiation of bone marrow stromal cells (BMSCs). (**a–g**) Representative alizarin red staining images demonstrating mineralization by murine BMSCs derived from the control groups (**a**) and osteoporoses induced by natural aging (**b**), accelerated senescence (SAMP6) (**c**), ovariectomy (OVX) (**d**), type 1 diabetes (T1D) (**e**), excessive glucocorticoids (GIOP) (**f**), and orchidectomy (ORX) (**g**). Mice were sacrificed after 4 weeks of modeling. 1^st^-passaged BMSCs were seeded at 2 × 10^5^ cells/well in 12-well plates and underwent osteogenic induction for 14 days. Bars: 5 mm. (**h–j**) The corresponding parameters of mineralized area over total area (**h**), number of mineralized nodules per well (**i**) and average size of nodules (**j**) showing decreased mineralization of BMSCs in osteoporoses except in GIOP. BMSCs from natural aging mice developed the least number of mineralized nodules. (**k**) After 14-day osteogenic induction, quantitative real-time polymerase chain reaction (qRT-PCR) analysis of the mRNA expression level of osteogenic marker gene *Runt-related transcription factor 2 (RUNX2)*. The corresponding values showed inhibited osteogenic differentiation of BMSCs in osteoporoses except in GIOP. BMSCs from the natural aging mice expressed the lowest level of *RUNX2*. The expression of *RUNX2* was normalized to that of *ACTIN*. Data represents mean ± standard deviation (SD). n = 6 per group. ^a^*P* < 0.05 with the control groups; ^b^*P* < 0.05 with the natural aging group; ^c^*P* < 0.05 with the SAMP6 group; ^d^*P* < 0.05 with the OVX group; ^e^*P* < 0.05 with the T1D group; ^f^*P* < 0.05 with the GIOP group; ^g^*P* < 0.05 with the ORX group.

These findings prompted us to further determine the changes and putative differences in multi-lineage differentiation capabilities of these BMSCs toward osteoblasts and adipocytes. In osteogenic induction, except those from GIOP mice, BMSCs showed reduction in mineralization capability, as revealed by alizarin red staining (Fig. 6a–g) and quantitative analyses on the area (Fig. 6h), number (Fig. 6i) and size (Fig. 6j) of the mineralized nodules. Notably, BMSCs derived from natural aging mice formed the lowest number of mineralized nodules. BMSCs from the GIOP model, however, did not exhibit changes of the mineralization potential (Fig. 6h–j). After 14-day osteogenic induction, qRT-PCR analysis on the mRNA expression level of osteogenic marker gene *Runt-related transcription factor 2 (RUNX2)* showed down-regulated expression in BMSCs derived from osteoporotic models except GIOP. The *RUNX2* level in BMSCs derived from natural aging mice declined the most (Fig. 6k). No sexual differences were detected between OVX and ORX models. The mineralization was comparable in the control groups of the 6 osteoporotic models (Supplementary Fig. S10).

### Adipogenic differentiation of BMSCs derived from different models

**Figure 7:**
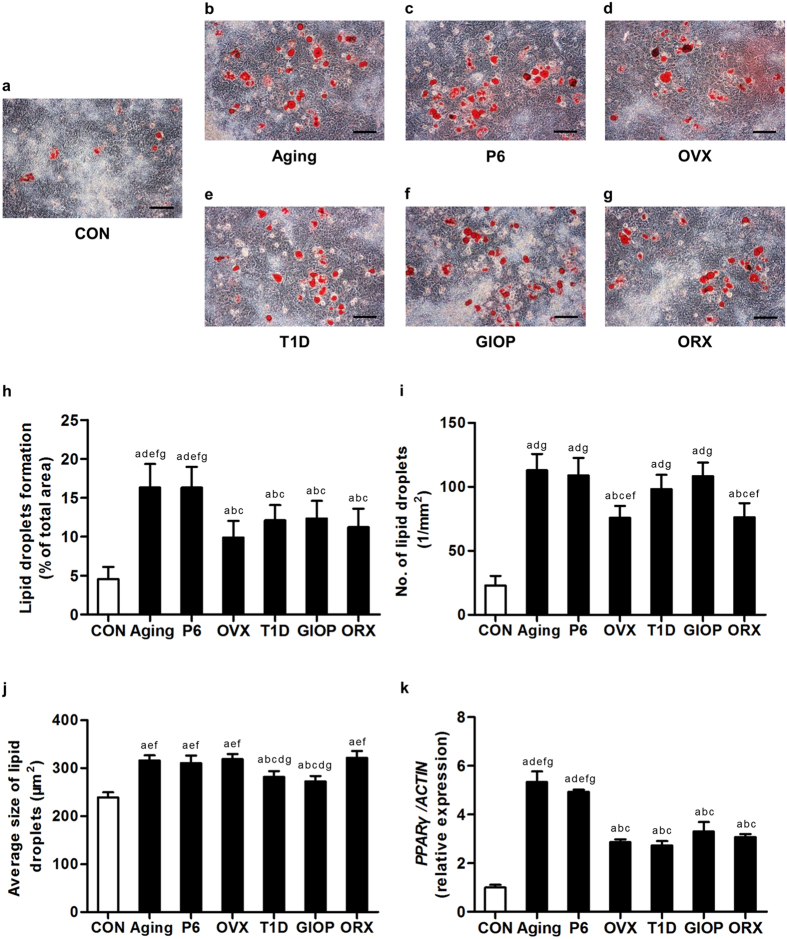
Adipogenic differentiation of bone marrow stromal cells (BMSCs). (**a–g**) Representative oil red O staining images demonstrating formation of lipid droplets by murine BMSCs derived from the control groups (**a**) and osteoporoses induced by natural aging (**b**), accelerated senescence (SAMP6) (**c**), ovariectomy (OVX) (**d**), type 1 diabetes (T1D) (**e**), excessive glucocorticoids (GIOP) (**f**), and orchidectomy (ORX) (**g**). Mice were sacrificed after 4 weeks of modeling. 1^st^-passaged BMSCs were seeded at 2 × 10^5^ cells/well in 12-well plates and underwent adipogenic induction for 14 days. Bars: 100 μm. (**h–j**) The corresponding parameters of lipid droplets formation over total area (**h**), number of lipid droplets per square millimeter (**i**) and average size of lipid droplets (**j**) showing stimulated adipogenic differentiation of BMSCs in all of the 6 types of osteoporoses. BMSCs from natural aging mice and the SAMP6 mice developed the largest area of lipid droplets. (**k**) After 14-day adipogenic induction, quantitative real-time polymerase chain reaction (qRT-PCR) analysis of the mRNA expression level of adipogenic marker gene *Peroxisome proliferator activated receptor gamma (PPARγ)*. The corresponding values showed increased adipogenic differentiation of BMSCs in all of the 6 types of osteoporoses. BMSCs from natural aging mice and the SAMP6 mice expressed the highest level of *PPARγ*. The expression of *PPARγ* was normalized to that of *ACTIN*. Data represents mean ± standard deviation (SD). n = 6 per group. ^a^*P* < 0.05 with the control groups; ^b^*P* < 0.05 with the natural aging group; ^c^*P* < 0.05 with the SAMP6 group; ^d^*P* < 0.05 with the OVX group; ^e^*P* < 0.05 with the T1D group; ^f^*P* < 0.05 with the GIOP group; ^g^*P* < 0.05 with the ORX group.

We next evaluated the adipogenic potential of BMSCs derived from the 6 osteoporotic models. As shown in Fig. 7a–g, BMSCs derived from all of the 6 models demonstrated increased adipogenic differentiation, forming more and larger lipid droplets compared to the control group. Quantitative analysis on the area (Fig. 7h), number (Fig. 7i) and size (Fig. 7j) of lipid droplets revealed significant increase of the adipogenic potential. Of note, BMSCs from natural aging mice and SAMP6 mice formed the most and the largest lipid droplets, while BMSCs from T1D and GIOP mice formed more but smaller lipid droplets compared to those from OVX and ORX mice. After 14-day adipogenic induction, qRT-PCR analysis on the mRNA expression level of adipogenic marker gene *Peroxisome proliferator activated receptor gamma (PPARγ)* showed up-regulated expression in BMSCs derived from all of the 6 osteoporotic models, among which the levels in BMSCs derived from natural mice and SAMP6 mice were the highest (Fig. 7k). No sexual differences were detected between OVX and ORX models. The adipogenic differentiation was paralleled in the control groups of the 6 osteoporotic models (Supplementary Fig. S11).

## Discussion

Local and systemic pathologic factors of bone synergistically induce bone loss and increased marrow adiposity in the development of osteoporosis^2^,^6^,^9^. Recent reports have shown that osteoblasts and marrow adipocytes may derive from different subpopulations of BMSCs *in vivo*^17^,^18^,^19^. However, niche factors regulate these heterogeneous mesenchymal progenitors as a whole to influence their direction of differentiation^4^,^15^,^22^. Previous studies have shown potential differences of bone and MAT phenotypes among osteoporoses of diverse pathologies and between sexes^11^,^12^,^13^,^14^, and demonstrated characteristics of specific BMSC subpopulations physiologically^17^,^18^,^19^. However, in differential pathologic states, pathological characteristics of BMSCs to osteoporoses and potential functional differences are unknown. In our study, through analyzing behaviors of BMSCs derived from 6 types of osteopenias of diverse pathologies, we revealed dysfunction of BMSCs in the common shift from osteoblastogenesis to adipogenesis in osteoporoses of diverse pathologies, but functional differences existed among different models, particularly in T1D and GIOP.

Many risk factors have been revealed in the development of senile osteoporosis, among which recent discoveries have shown that inflammation is of key importance in mediating disordered bone remodeling^2^. Similarly, estrogen deficiency exerts pathologic effects on bone through immunologically mediated mechanisms in postmenopausal osteoporosis^4^,^43^. Previously, we have reported that proinflammatory cytokines tumor necrosis factor-alpha (TNF-α) and interferon-gamma (IFN-γ) synergistically induced impairments in the self-renewal and osteogenesis of BMSCs from OVX mice^6^. We have also demonstrated that systemic administration of TNF-α neutralized antibody in OVX mice rescued the osteogenic defect of BMSCs^4^. However, aging differs from postmenopause in that aging directly provokes cell-intrinsic dysfunction like telomere attrition in the age-related functional decline of BMSCs^21^. In this study, we showed that natural aging mice developed more severe bone loss compared to OVX mice and the accelerated senescent SAMP6 mice, despite that SAM strains have a different background of AKR/J^25^. BMSCs derived from natural aging mice contained higher percentages of senescent cells and had weaker capability of mineralization. These findings highlighted that bone and BMSCs underwent more damages by the interactions of complicated pathologic factors during the natural aging process. A recent study have reported that age-related osteopenia in Sca1^−/−^ mice could be rescued by delivery of exogenous BMSCs to replenish the endogenous BMSC pool^44^, suggesting feasibility of targeting resident BMSCs in the regenerative therapy for osteoporosis.

Hypogonadism is considered to be one of the major risk factors for osteoporosis in human^45^,^46^,^47^. Although osteoporoses in men and women is similar, it has been established that postmenopausal women had higher amount of MAT than aged men after adjustment for BMD^11^,^13^. While the pathogenesis of postmenopausal osteoporosis is well known, the mechanisms underlying bone loss after withdrawal of androgens are less understood. Studies have shown that both estrogen and androgen therapies prevented bone loss and MAT accumulation in rodents^46^,^47^. It is now accepted that androgen exerts skeletal effects directly or by being metabolized into estrogens through the enzyme aromatase^5^,^45^. Leskela *et al*. showed that estrogen treatment promoted osteogenic differentiation of BMSCs from both female and male donors, but androgen treatments had no effect in either sex, suggesting potential differences of BMSCs in response to different sex hormones^48^. However, we did not detect sexual difference between OVX and ORX models in bone and BMSCs, indicating changes in the bone metabolism in male were highly reminiscent of the changes in female under sex hormone deficiency in the present study.

Secondary bone loss is induced by an underlying cause such as excessive glucocorticoids and diabetes^8^,^49^. DEX is widely used as a component in both osteogenic and adipogenic inducing media of mesenchymal progenitors *in vitro*^3^,^36^,^37^. In GIOP, Li *et al*. reported that excessive DEX injection shifted BMSC differentiation to adipocytes. Notably, they induced osteogenic differentiation of *ex vivo* BMSCs under only bone morphogenetic protein 2 (BMP2), but they added 1 μM DEX during adipogenic induction^22^. *In vitro* studies have demonstrated dose-dependent effects of DEX on BMSC differentiation^36^,^38^. Walsh *et al*. showed that at a physiologically equivalent concentration of 10 nM, DEX promoted osteogenic differentiation of BMSCs^38^. However, at concentrations of over 100 nM, effects of DEX on the adipogenic potential overweighed those on the osteogenic potential^36^,^50^. In this study, we added 10 nM DEX into osteogenic inducing media and 500 nM DEX into adipogenic inducing media as reported^29^. We identified that the mineralization of BMSCs derived from GIOP mice was not significantly inhibited, while the adipogenic potential increased. Works by Aubin *et al*. pointed out that the mesenchymal progenitors in bone marrow could be divided into two populations of precursors: (1) the committed progenitors, which are capable of constitutive differentiation *in vitro* in standard culture conditions; (2) the inducible progenitors, which undergo osteoblastic differentiation under specific inductive stimuli like DEX^51^. In our culture conditions and *in vivo*, it is probable that both committed and inducible osteoblastic progenitors existed. The reduced bone formation should be attributed to detrimental effects of DEX on osteoblast lineage cells, inducing apoptosis and inhibiting proliferation, as shown here and before^3^,^35^,^49^.

T1D is a chronic autoimmune metabolic disease characterized by hypoinsulinemia, hyperglycemia and various complications including osteoporosis^7^,^52^. It has been reported that patients with T1D developed increased marrow adiposity^33^. As far as we know, there are no reports on adipogenic potential of BMSCs in T1D, in which environmental factors like inflammation and advanced glycation end products (AGEs) may exert detrimental impacts on BMSC function^20^,^53^. Studies have shown that osteogenic potential of BMSCs decline in STZ-treated rats, while the results on BMSC proliferation were controversial^20^,^34^,^54^. Zhao *et al*. documented impaired proliferation of BMSCs after rats modeling for 12 weeks^34^,^54^. Stolzing *et al*. further reported that colony-forming capability of rat BMSCs increased after 4-week modeling but decreased after 12 weeks^20^. STZ-induced hyperglycemia has also been shown to increase cellularity in other forms of mesenchyme-derived connective tissues, e.g. the cardiac and renal fibrosis^55^. In our study, we established T1D by multiple low dose of STZ, as it closely replicates the damages of pancreatic β-cells, an activation of the immune system and many of the complications seen in human T1D^56^,^57^. We clarified that BMSCs derived from T1D mice showed slight elevation in CFE and proliferation in the 4-week period. The impaired osteogenic potential and the misdirected differentiation to adipocytes constituted the major dysfunction of BMSCs in T1D-induced osteoporosis.

In summary, we uncovered differential BMSC characteristics in the common shift from osteoblastogenesis to adipogenesis in osteopenias of diverse pathologies. These findings might improve the understanding of the pathophysiological contributions of BMSCs to the bone metabolism, and provide theoretical basis for the functional modulation of resident BMSCs in the regenerative therapy for osteoporosis.

## Methods

### Animals

Animal experiments were approved by Fourth Military Medical University and were performed following the Guidelines of Intramural Animal Use and Care Committee of Fourth Military Medical University. For the natural aging group (n = 6), 17-month-old female C57BL/6J mice were used (weight, 28–30 g) with a 2-month-old female control (n = 6) (weight, 20–22 g)^1^. The mice were modeled for 1 month and were sacrificed at 18-month old and 3-month old, respectively. The below mice of other groups were used at 2-month old, modeled for 1 month, and sacrificed at 3-month old. For the accelerated senescence group (n = 6) and its control (n = 6), female SAMP6 mice and female senescence-accelerated mice-resistant 1 (SAMR1) mice^39^ were used (weight, 20–22 g), which were AKR/J background and were obtained from our colony provided by the Council for SAM Research of Kyoto University, Japan. For the OVX, T1D, GIOP and ORX groups and their control groups, C57BL/6J mice were used (weight, 20–22 g). For the OVX group (n = 6) and its Sham control (n = 6), female mice underwent either a bilateral OVX or a Sham operation by the dorsal approach under general anesthesia^6^. For the T1D group (n = 6) and its control (n = 6), female mice accepted either 50 mg/kg/d multiple low dose of STZ (Sigma-Aldrich, St. Louis., MO, USA) for 5 consecutive days (1 injection/d) dissolved in about 200 μl 0.1 M citrate buffer (pH 4.5) or equivalent citrate buffer through intraperitoneal injection. After STZ injections, the concentrations of non-fasting blood glucose were measured every 3 days using a glucometer (Roche Bioproducts, Basel, Switzerland) following tail vein-puncture whole blood sampling and the mice with over 11.1 mmol/L glucose in blood were classified as hyperglycemic^40^. Any mice whose blood glucose was not maintained over 11.1 mmol/L throughout the experimental period were excluded, and 6 hyperglycemic mice were finally included. For the GIOP group (n = 6) and its control (n = 6), female mice accepted either 20 mg/kg/d dextran-conjugated water-soluble DEX (Sigma-Aldrich, St. Louis., MO, USA) dissolved in about 200 μl PBS or equivalent PBS for 28 consecutive days (1 injection/d) through intraperitoneal injection^41^. For the ORX group (n = 6) and its Sham control (n = 6), male mice underwent either a bilateral ORX or a Sham operation by the abdominal approach under general anesthesia^42^. The mice were maintained with good ventilation and a 12-h light/dark cycle, and were kept feeding and drinking ad libitum. Intraperitoneal injections were performed via the right lower quadrant of the abdominal area, 1 cm away from the midabdominal line. Mice were kept at head-down position to ensure that intraperitoneally injected fluid was not accidentally placed in intestine.

### Micro-CT analysis

For trabecular bone mass evaluation, a desktop micro-CT system (eXplore Locus SP, GE Healthcare, USA) was employed. At sacrifice, the left femora were removed, fixed overnight in 4% paraformaldehyde, and scanned at a resolution of 8 μm, a voltage of 80 kV, and a current of 80 μA. The micro-CT system was calibrated with a standard hydroxyapatite to enable accurate analysis for bone mass. Trabecular bone data were obtained at a region of interest (ROI) in the distal metaphysis. The ROI was defined as a cylinder area located along the long axis of femur, with the same size in each specimen. The intersection point of the median sagittal plane, the median coronal plane and the epiphysis of femur was set as the landmark. The ROI was 0.3 mm to 1.3 mm away from the landmark. In the median sagittal plane, the lateral margins were 0.5 mm away from the long axis at both sides. In the median coronal plane, the lateral margins were 0.6 mm away from the long axis at both sides. Data were analyzed with the Micview V2.1.2 software, and the quantification was performed using the parameters of BMD, bone volume over tissue volume (BV/TV), trabecular number (Tb.N), trabecular thickness (Tb.Th) and trabecular separation (Tb.Sp)^4^.

### Bone histomorphometric analysis

For trabecular bone and MAT histomorphometric analysis, after micro-CT analysis, the femora were decalcified with 10% EDTA (pH, 7.2–7.4) and embedded in paraffin. 5-mm-thick sagittal serial sections of proximal metaphyses were prepared (RM2125, Leica, Germany). The sections were further deparaffinized and stained with H&E. Percentages of trabecular bone area and number and area of marrow adipocytes (identified as empty oval spaces) were calculated using the ImageJ 1.47 software in the area of interest from at least 5 consecutive microscopic fields^1^. The quantification was performed based on semi-automatic plug-ins for recording and repeating same operation steps. The thresholds for distinguishing the quantification objectives with the background were set based on preliminary tests with several differential photographs.

For bone formation examination, double calcein labeling was performed according to previous studies with minor modifications^4^,^58^. At 16 d and 2 d prior to sacrifice, mice received double intraperitoneal injection of 20 mg/kg calcein (Sigma-Aldrich, USA). Calcein was dissolved at a concentration of 2 mg/ml in PBS supplemented with 1 mg/ml NaHCO_3_ (Sigma-Aldrich, USA), and was injected at 10 μl/g each time away from light. Intraperitoneal injections were performed via the right lower quadrant of the abdominal area, 1 cm away from the midabdominal line. Mice were kept at head-down position to ensure that intraperitoneally injected fluid was not accidentally placed in intestine. At sacrifice, right femora were isolated, fixed in 80% ethanol, and embedded in methyl methacrylate. The specimens were sagittally sectioned into 30-μm sections using a hard tissue slicing machine (SP1600, Leica, Germany) away from light. Both double-labeled and single-labeled cortical endosteum and trabecular surfaces were evaluated by a fluorescence microscope (STP6000, Leica, Germany) with an excitation wavelength of 488 nm. Quantification was performed based on at least 5 photographs using the parameters of MAR and MS/BS. BFR was calculated as MAR × MS/BS, according to previous studies^58^.

### Isolation and culture of murine BMSCs

Isolation and culture of murine BMSCs were performed as previously described^29^. Briefly, murine bone marrow cells were harvested from tibiae and humeri and were seeded into 9-cm culture dishes. After incubation for 24 hours at 37 °C, non-adherent cells were removed by rinsing with PBS. The adherent cells were cultured with α-MEM supplemented with 20% fetal bovine serum (FBS), 2 mM L-glutamine, 100 U/ml penicillin, and 100 g/mL streptomycin in a humidified atmosphere of 5% CO_2_ at 37 °C, and the media were changed every 2 days. At confluence, BMSCs were passaged with 0.25% trypsin. All of the reagents were from Invitrogen (Carlsbad, CA, USA). Approximately 1.2~2.4 × 10^6^ 1^st^-passaged BMSCs could be obtained from a single mouse, and the cells used in the various assays were derived from the same mouse.

### Flow cytometry analysis

For analysis of the surface makers^29^,^59^, 1^st^ passaged BMSCs derived from C57BL/6J mice were suspended in PBS supplemented with 3% FBS at 1 × 10^6^ cells/ml. 2 × 10^5^ cells/tube were added with 1-μl FITC-conjugated anti-mouse CD11b antibody, 1-μl PE-conjugated anti-mouse CD29 antibody, 1-μl PE-conjugated anti-mouse CD34 antibody, 1-μl PE-conjugated anti-mouse CD45 antibody, 1-μl PE-conjugated anti-mouse CD106 antibody, and 1-μl FITC-conjugated anti-mouse SCA-1 antibody (all from Abcam, UK). Non-immune immunoglobulin of the same isotype was used as the negative control. BMSCs were incubated in 4 °C for 30 min in dark, and then washed twice with PBS supplemented with 3% FBS. The percentages of positively stained cells were determined with a flow cytometer (FACSAria, BD, USA) equipped with the FACSDiva Version 6.1.3 software.

For analysis of apoptosis^60^, 1^st^ passaged BMSCs were harvested 3 days after seeding and evaluated by FITC-conjugated Annexin V and propidium iodide (PI) double staining according to the manufacturer’s instruction of Annexin V Apoptosis Detection Kit I (BD Biosciences). After incubation, cell apoptosis was detected with a flow cytometer (FACSAria, BD, USA) equipped with the FACSDiva Version 6.1.3 software. The percentages of early apoptotic (FITC^+^PI^−^) plus late apoptotic/necrotic (FITC^+^PI^+^) cells were expressed as apoptotic BMSC percentages, as stated^60^.

### CFE analysis

CFE assay was conducted as stated before^20^. After treated with ACK lysis buffer (Lonza, Basel, Switzerland) to remove red blood cells, murine primary bone marrow cells were plated in 5-cm culture dishes at a density of 1 × 10^5^ cells/cm^2^ and cultured. The formation of colonies was evaluated after 14 days of culture. The colonies were fixed with 4% paraformaldehyde for 30 min and stained with crystal violet for 5 min. Colonies with over 50 cells were counted and the average size of the colonies were evaluated using the ImageJ 1.47 software. The quantification was performed based on semi-automatic plug-ins for recording and repeating same operation steps. The thresholds for distinguishing the quantification objectives with the background were set based on preliminary tests with several differential photographs.

### MTT assay for proliferation rate analysis

BMSCs at the 1^st^ passage were plated at 2 × 10^3^ cells/well in 96-well plates. At the same time point in Day-1, 3, 5, 7 and 9 (3 wells per time point), cells were incubated with 20-μl 5 mg/ml MTT (MP Biomedicals, Irvine, CA, USA) for 4 h. The precipitates were extracted with 180-μl DMSO and the absorbance was measured at the optical density (OD) of 490 nm. The proliferation was depicted by the OD_490_ curves and the statistical analysis was performed between the control group and each of the osteoporotic groups. At Day-9, intergroup comparisons of the cell viability among the osteoporotic models were further statistically analyzed.

### SA-β-gal assay for cellular senescence analysis

β-gal activity was determined in the 1^st^-passaged BMSCs using the Senescence β-Galactosidase Staining Kit (Beyotime, Shanghai, China), according to the manufacturer’s instructions^39^. Cells were plated at 1 × 10^5^ cells/well in 12-well plates. The percentage of SA–β-gal positive cells was determined from at least 5 fields of view using the ImageJ 1.47 software. The quantification was performed based on semi-automatic plug-ins for recording and repeating same operation steps. The thresholds for distinguishing the quantification objectives with the background were set based on preliminary tests with several differential photographs.

### Osteogenic differentiation

To induce osteogenic differentiation, BMSCs at the 1^st^ passage were cultured in osteogenesis-inducing media containing 100 μg/ml ascorbic acid (MP Biomedicals, Irvine, CA, USA), 2 mM β-glycerophosphate (Sigma-Aldrich, St. Louis., MO, USA) and 10 nM DEX (Sigma-Aldrich, St. Louis., MO, USA). Cells were plated at 2 × 10^5^ cells/well in 12-well plates. The media were changed every 3 days. After induction for 14 days, alizarin red staining was performed to determine the mineralization^6^. Quantitative parameters of the percentage of the mineralized area and the number and the average size of mineralized nodules were determined with the ImageJ 1.47 software. The quantification was performed based on semi-automatic plug-ins for recording and repeating same operation steps. The thresholds for distinguishing the quantification objectives with the background were set based on preliminary tests with several differential photographs.

### Adipogenic differentiation

To induce adipogenic differentiation, BMSCs at the 1^st^ passage were cultured in adipogenesis-inducing media containing 0.5 mM isobutylmethylxanthine (MP Biomedicals, Irvine, CA, USA), 0.5 μM DEX (Sigma-Aldrich, St. Louis., MO, USA) and 60 mM indomethacin (MP Biomedicals, Irvine, CA, USA). Cells were plated at 2 × 10^5^ cells/well in 12-well plates. The media were changed every 3 days. After induction for 14 days, oil red O staining was performed to determine the lipid droplet formation in adipogenesis^1^. Quantitative parameters of the percentage of the area, the number and the average size of lipid droplets were determined from at least 5 fields of view with the ImageJ 1.47 software. The quantification was performed based on semi-automatic plug-ins for recording and repeating same operation steps. The thresholds for distinguishing the quantification objectives with the background were set based on preliminary tests with several differential photographs.

### qRT-PCR analysis

Total RNA was collected from the 1^st^-passaged BMSCs (1 × 10^5^ cells/well in 12-well plates) and after 14-day induction for multilineage differentiation (2 × 10^5^ cells/well in 12-well plates). RNA was extracted by the direct addition of Trizol Reagent (Takara, Tokyo, Japan) to the culture plate and purified by phenol–chloroform extraction. cDNA synthesis and PCR procedures were performed as described^29^. The primer sequences of the genes detected in this study were listed in the Supplementary information (Supplementary Table S1). Relative expression level of each gene was obtained by normalizing against *ACTIN* abundance.

### Statistical analysis

All the results are represented as the mean ± standard deviation (SD). The data were analyzed using One-way analysis of variance (ANOVA) followed by the post-hoc test of Newman-Keuls Multiple Comparison in the GraphPad Prism 5.01 software. Values of *P* < 0.05 were considered to be statistically significant.

## Additional Information

**How to cite this article**: Sui, B. *et al*. Mesenchymal progenitors in osteopenias of diverse pathologies: differential characteristics in the common shift from osteoblastogenesis to adipogenesis. *Sci. Rep.* **6**, 30186; doi: 10.1038/srep30186 (2016).

## Supplementary Material

Supplementary Information
