# Supplementary material for: Mesenchymal progenitors in osteopenias of diverse pathologies: differential characteristics in the common shift from osteoblastogenesis to adipogenesis
Source: Sci Rep. 2016 Jul 22;6:30186. doi: 10.1038/srep30186 (PMC4957106; doi:10.1038/srep30186)
Supplement: Supplementary Information [file srep30186-s1.pdf]

Mesenchymal progenitors in osteopenias of diverse pathologies: differential features in the common shift from osteoblastogenesis to adipogenesis

Bingdong Sui[1, 2†], Chenghu Hu[1, 2†], Li Liao[1, 2], Yichen Chen[1, 2], Xinyi Zhang[1, 2], Xin Fu[1, 2], Chenxi Zheng[1, 2], Meng Li[1], Ling Wu[1, 2], Xinyi Zhao[3,4] & Yan Jin[1, 2*]

[1]State Key Laboratory of Military Stomatology, Center for Tissue Engineering, Fourth Military Medical University, Xi'an 710032, Shaanxi, China.

[2]Xi'an Institute of Tissue Engineering and Regenerative Medicine, Xi'an 710032, Shaanxi, China.

[3]Department of Dental Materials, School of Stomatology, Fourth Military Medical University, Xi'an 710032, Shaanxi, China.

[4]State Key Laboratory of Military Stomatology, Department of Dental Materials, Fourth Military Medical University, Xi'an 710032, Shaanxi, China.

[†]These authors contributed equally to this work.

[*]Correspondence and requests for materials should be addressed to Prof. Yan Jin

State Key Laboratory of Military Stomatology, Center for Tissue Engineering, Fourth Military Medical University, No. 145 West Changle Road, Xi'an, Shaanxi 710032, China. *E-mail:* yanjin@fmmu.edu.cn; *Tel:* +86-029-84776472; *Fax:* +86-029-83218039.

Journal: Scientific Reports.

Supplementary information: 11 figures and 1 table.

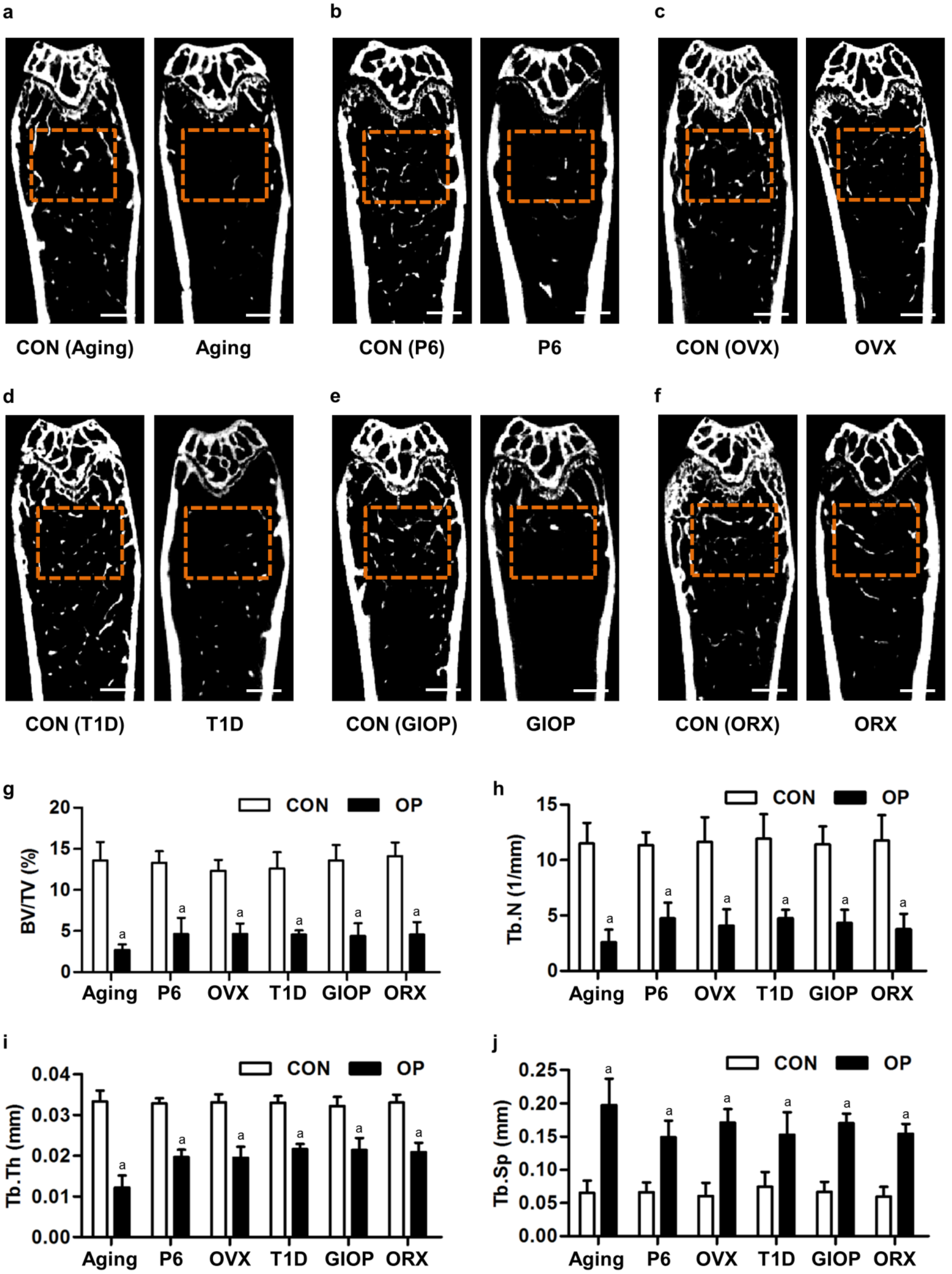

Supplementary Figure S1. The establishment of 6 different types of osteoporoses in mice. (**a-f**) Representative micro-CT images of the distal femora demonstrating successful establishment of 6 different

types of osteoporoses in mice. Mice were modeled 4 weeks for the control groups and osteoporoses induced by natural aging (**a**), accelerated senescence (SAMP6) (**b**), ovariectomy (OVX) (**c**), type 1 diabetes (T1D) (**d**), excessive glucocorticoids (GIOP) (**e**) and orchidectomy (ORX) (**f**). The orange boxes indicated the analyzed region of interest (ROI). Bars: 500 μm. (**g**) Analyzed by micro-CT, the parameter of bone volume over tissue volume (BV/TV) in the ROI demonstrating bone loss in the 6 types of osteoporoses. (**h-j**) Corresponding trabecular parameters of trabecular number (Tb.N) (**h**), trabecular thickness (Tb.Th) (**i**) and trabecular separation (Tb.Sp) (**j**) showing reduction of trabecular bone mass in 6 different types of osteoporoses. Data represents mean ± standard deviation (SD). n=6 per group. $^{a}P < 0.05$ with the control groups.

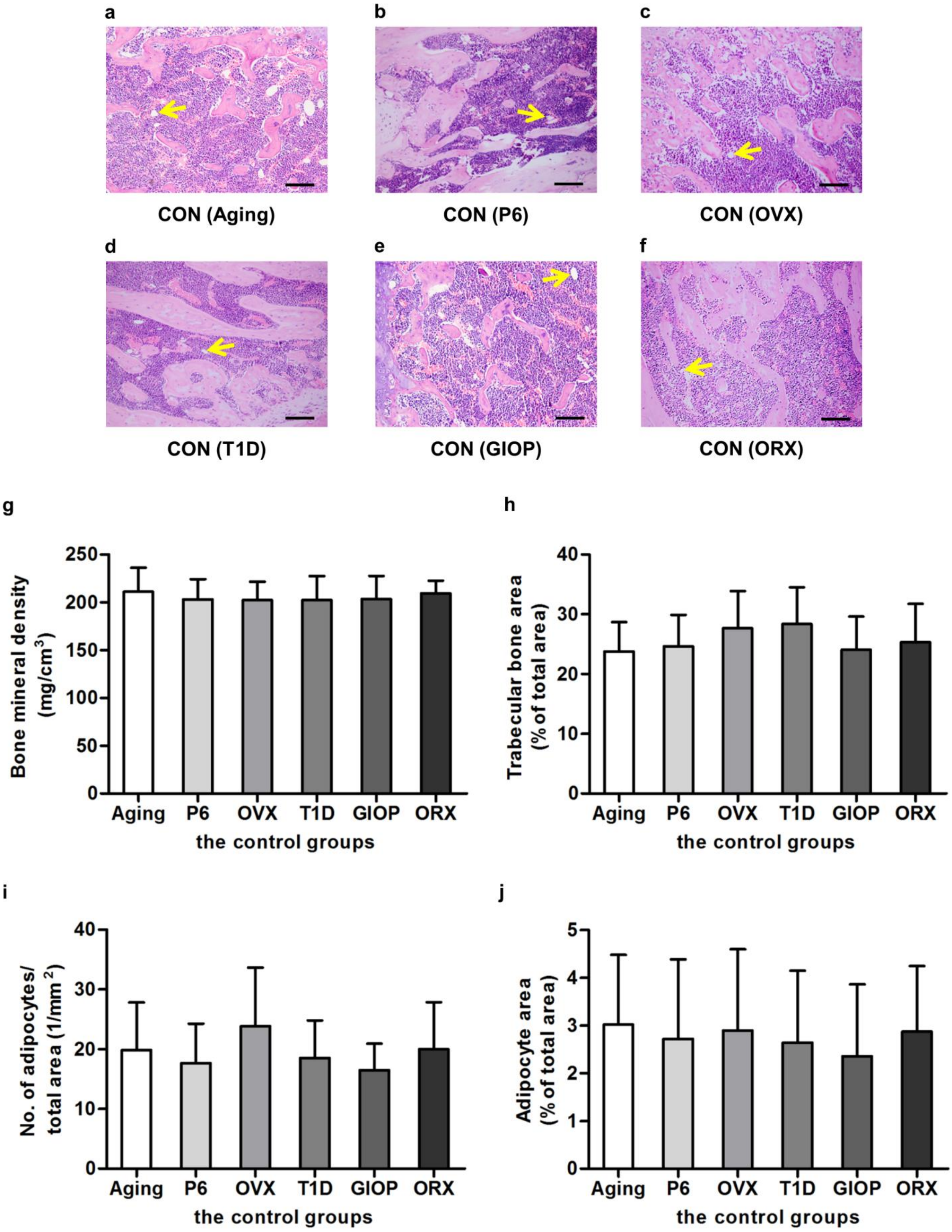

Supplementary Figure S2. Trabecular bone mass and marrow adiposity of the control groups. (**a-f**) Representative H&E images demonstrating trabecular bone and marrow adipose tissue in the distal femora of mice. Mice were modeled 4 weeks for the control groups of natural aging (**a**), accelerated senescence (SAMP6)

(**b**), ovariectomy (OVX) (**c**), type 1 diabetes (T1D) (**d**), excessive glucocorticoids (GIOP) (**e**) and orchidectomy (ORX) (**f**). The unstained area in the bone marrow represented empty spaces occupied by adipocytes with yellow arrows indicating. Bars: 100 μm. (**g**) Analyzed by micro-CT, the parameter of bone mineral density (BMD) in the distal metaphysis of femora demonstrating comparable bone mass in control groups of the 6 types of osteoporoses. (**h-j**) In bone histomorphometric analysis by H&E staining, the corresponding parameters of trabecular bone area over total area (**h**), number of adipocytes over total area (**i**) and adipocyte area over total area (**j**) showing paralleled bone mass and adipocytes in control groups of 6 different types of osteoporoses. Data represents mean ± standard deviation (SD). n=6 per group.

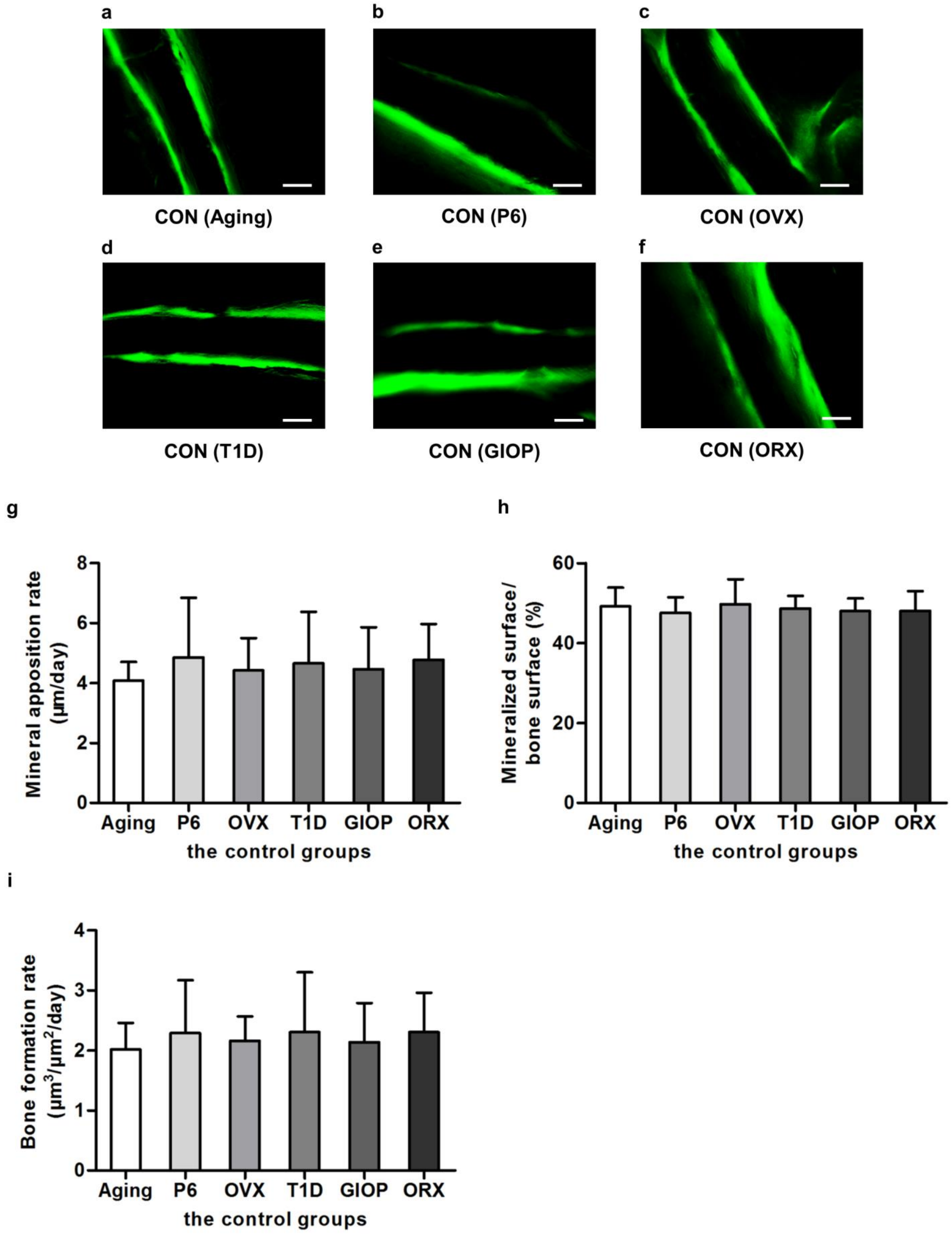

Supplementary Figure S3. Cortical bone formation parameters of the control groups. (**a-f**) Representative calcein labeling images demonstrating bone formation in the cortical endosteum of the distal femora of mice. Mice were modeled 4 weeks for the control groups of natural aging (**a**), accelerated

senescence (SAMP6) (**b**), ovariectomy (OVX) (**c**), type 1 diabetes (T1D) (**d**), excessive glucocorticoids (GIOP) (**e**) and orchidectomy (ORX) (**f**). Mice accepted double intraperitoneal injection of 20 mg/kg calcein at 16 days and 2 days prior sacrifice. Calcein was dissolved in PBS at 2 mg/ml supplemented with 1 mg/ml $NaHCO_3$. Bars: 100 μm. (**g-i**) The corresponding parameters of mineral apposition rate (**g**), mineralized surface over bone surface (**h**) and bone formation rate (**i**) showing comparable cortical bone formation in control groups of the 6 different types of osteoporoses. Data represents mean ± standard deviation (SD). n=6 per group.

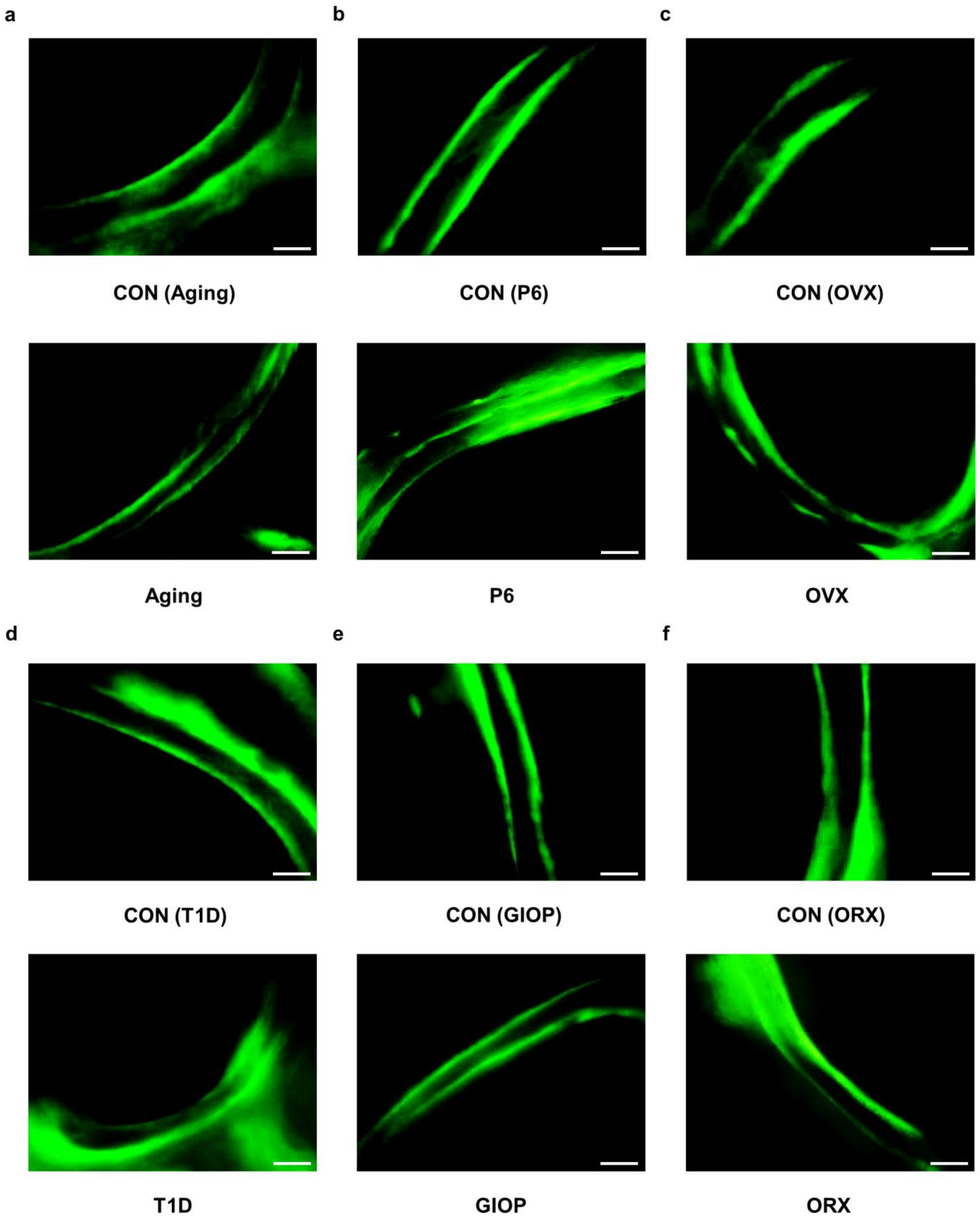

Supplementary Figure S4. Trabecular bone formation parameters. (**a-f**) Representative calcein labeling images demonstrating bone formation around the trabeculae of the distal femora of mice. Mice were modeled 4 weeks for the control groups and osteoporoses induced by natural aging (**a**), accelerated senescence (SAMP6)

(**b**), ovariectomy (OVX) (**c**), type 1 diabetes (T1D) (**d**), excessive glucocorticoids (GIOP) (**e**) and orchidectomy (ORX) (**f**). Mice accepted double intraperitoneal injection of 20 mg/kg calcein at 16 days and 2 days prior sacrifice. Calcein was dissolved in PBS at 2 mg/ml supplemented with 1 mg/ml $NaHCO_3$. Bars: 100 μm.

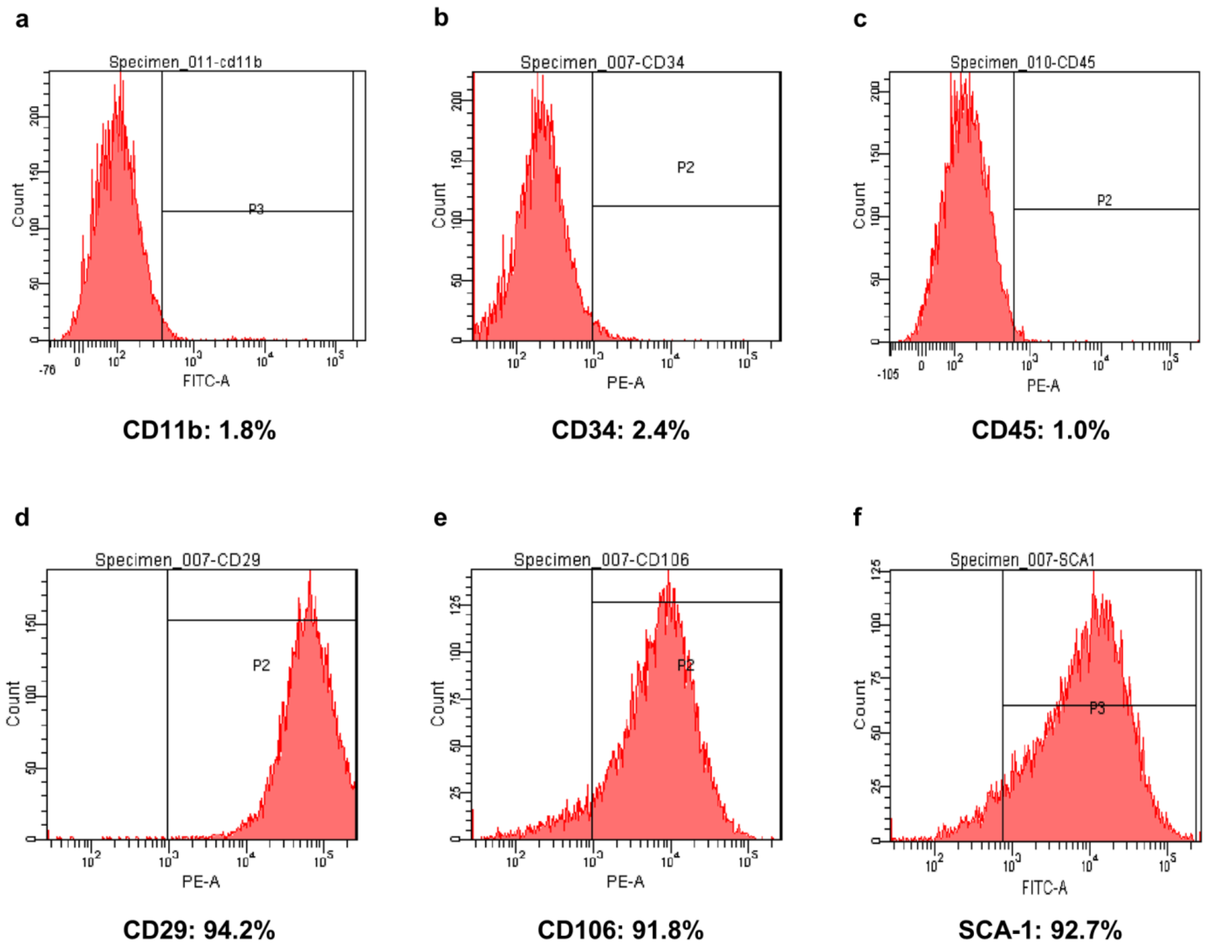

Supplementary Figure S5. Identification of surface markers of bone marrow stromal cells (BMSCs) used in the present study was in accordance with currently accepted standards. (**a-f**) Representative flow cytometric images demonstrating surface marker expression on BMSCs. BMSCs expressed surface makers considered to represent mesenchymal stem cells, including CD29, CD106 and stem cell antigen-1 (SCA-1), but were negative for hematopoietic markers CD11b, CD34 and CD45.

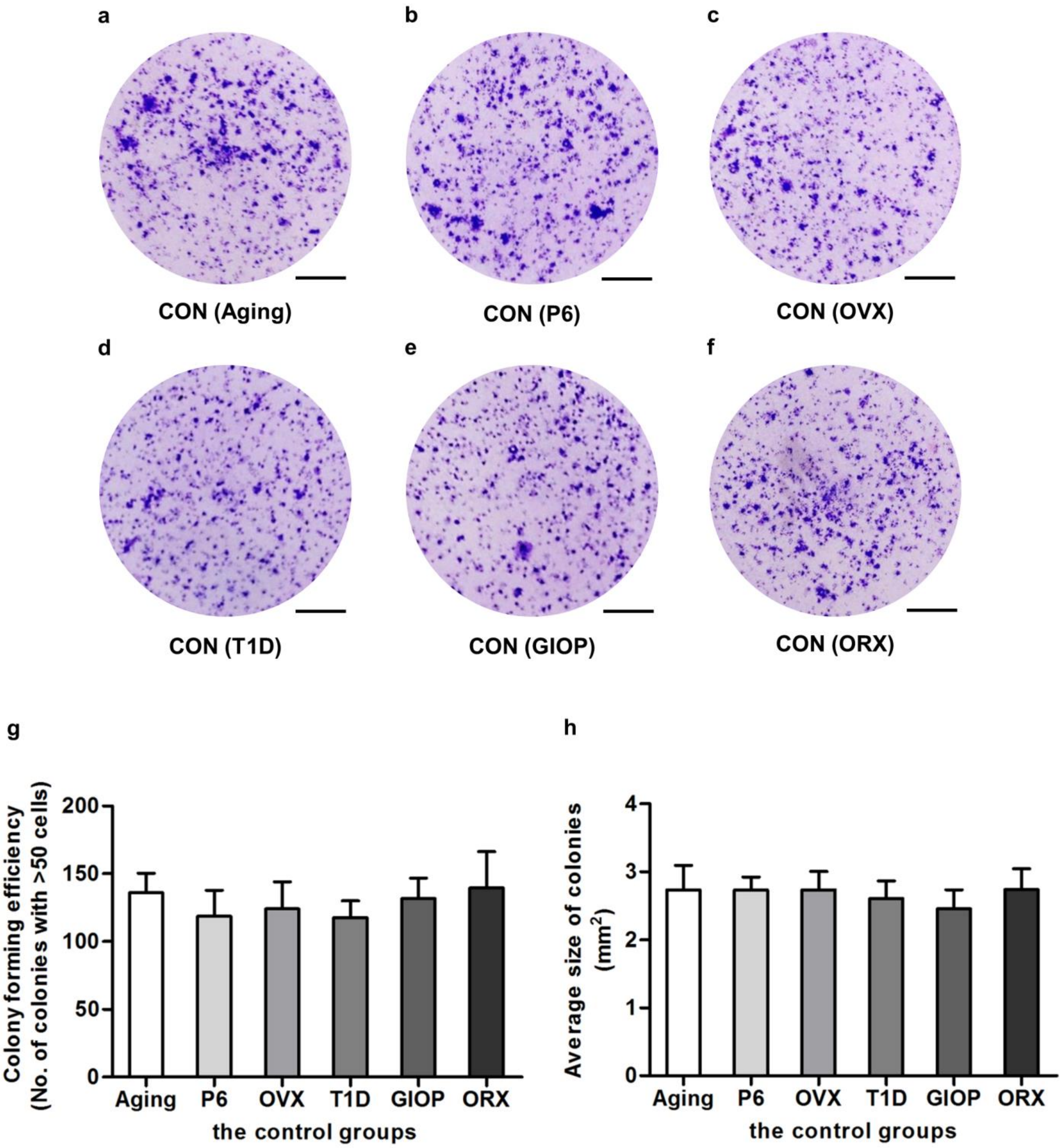

Supplementary Figure S6. Colony-forming efficiency of bone marrow stromal cells (BMSCs) derived from the control groups. (**a-f**) Representative images demonstrating colonies formed by primary murine BMSCs derived from the control groups of natural aging (**a**), accelerated senescence (SAMP6) (**b**), ovariectomy (OVX) (**c**), type 1 diabetes (T1D) (**d**), excessive glucocorticoids (GIOP) (**e**) and orchidectomy (ORX) (**f**). Mice were sacrificed after 4 weeks of modeling. Primary bone marrow cells were seeded at 1×10^5 cells/cm^2 after lysis of red blood cells. Bars: 1 cm. (**g, h**) The corresponding parameters of number of colonies

(**g**) and average size of colonies (**h**) showing paralleled colony-forming efficiency of BMSCs in control groups of the 6 different types of osteoporoses. Colonies with over 50 cells were taken into consideration. Data represents mean ± standard deviation (SD). n=6 per group.

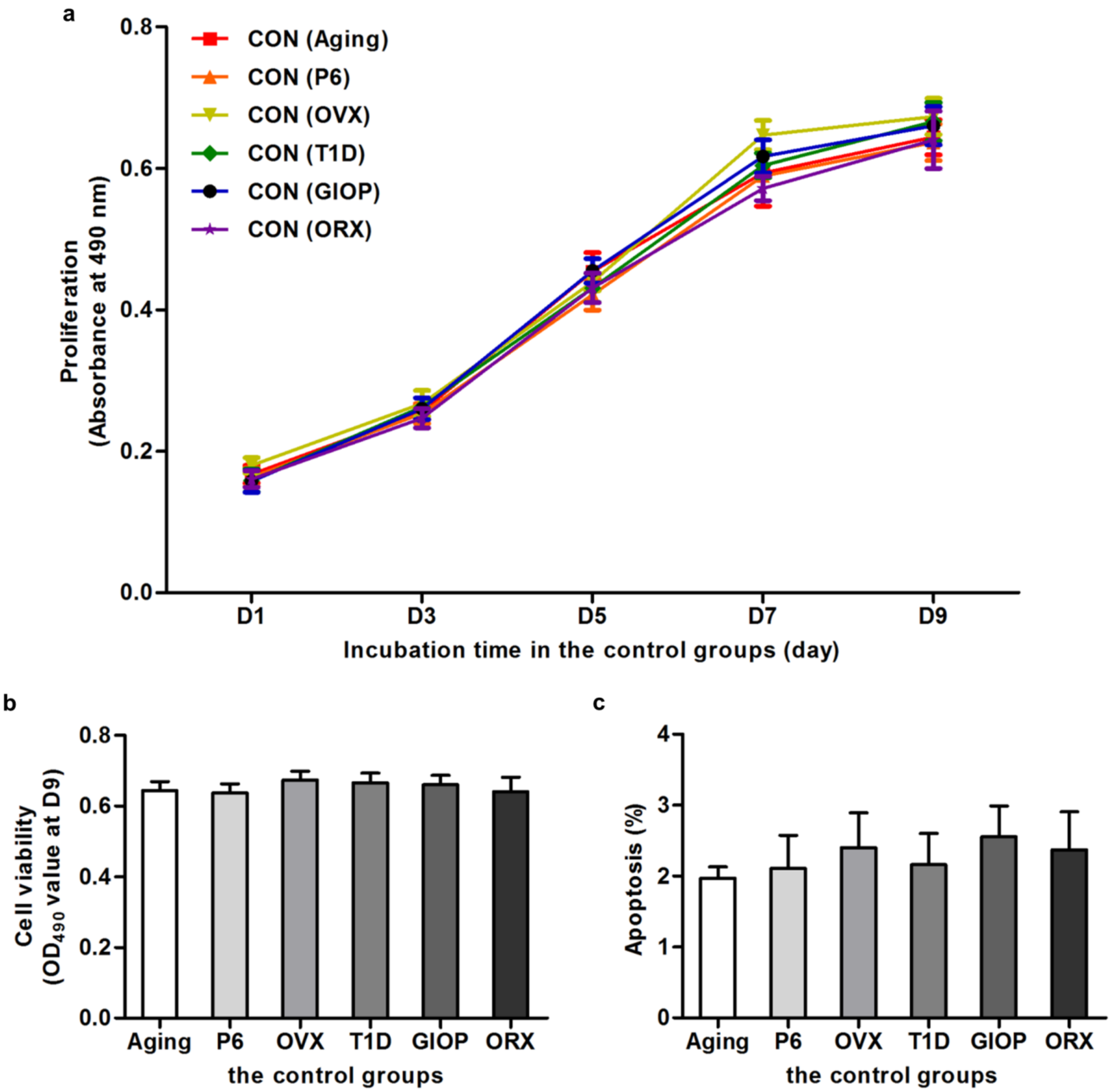

Supplementary Figure S7. Proliferation rate and apoptosis of bone marrow stromal cells (BMSCs) derived from the control groups. (**a**) Representative MTT curves demonstrating proliferation of murine BMSCs derived from the control groups of natural aging, accelerated senescence (SAMP6), ovariectomy (OVX), type 1 diabetes (T1D), excessive glucocorticoids (GIOP) and orchidectomy (ORX). Mice were sacrificed after 4 weeks of modeling. 1st-passaged BMSCs were seeded at 2×10^3 cells/well in 96-well plates and the cell viability was evaluated at the same time points in Day-1, 3, 5, 7, and 9. (**b**) The values of optical density at 490 nm in the MTT test at Day-9 showing similar proliferation rate of BMSCs in the control groups. (**c**)

Flow cytometry analysis showed similar apoptosis of BMSCs in the control groups. 1st passaged BMSCs were harvested 3 days after seeding and evaluated by FITC-conjugated Annexin V and propidium iodide (PI) double staining, and the percentages of early apoptotic ($FITC^+PI^-$) plus late apoptotic/necrotic ($FITC^+PI^+$) cells were expressed as apoptotic BMSC percentages. Representative flow cytometry plots for the controls were shown in Supplementary Figure S7. Data represents mean ± standard deviation (SD). n=6 per group.

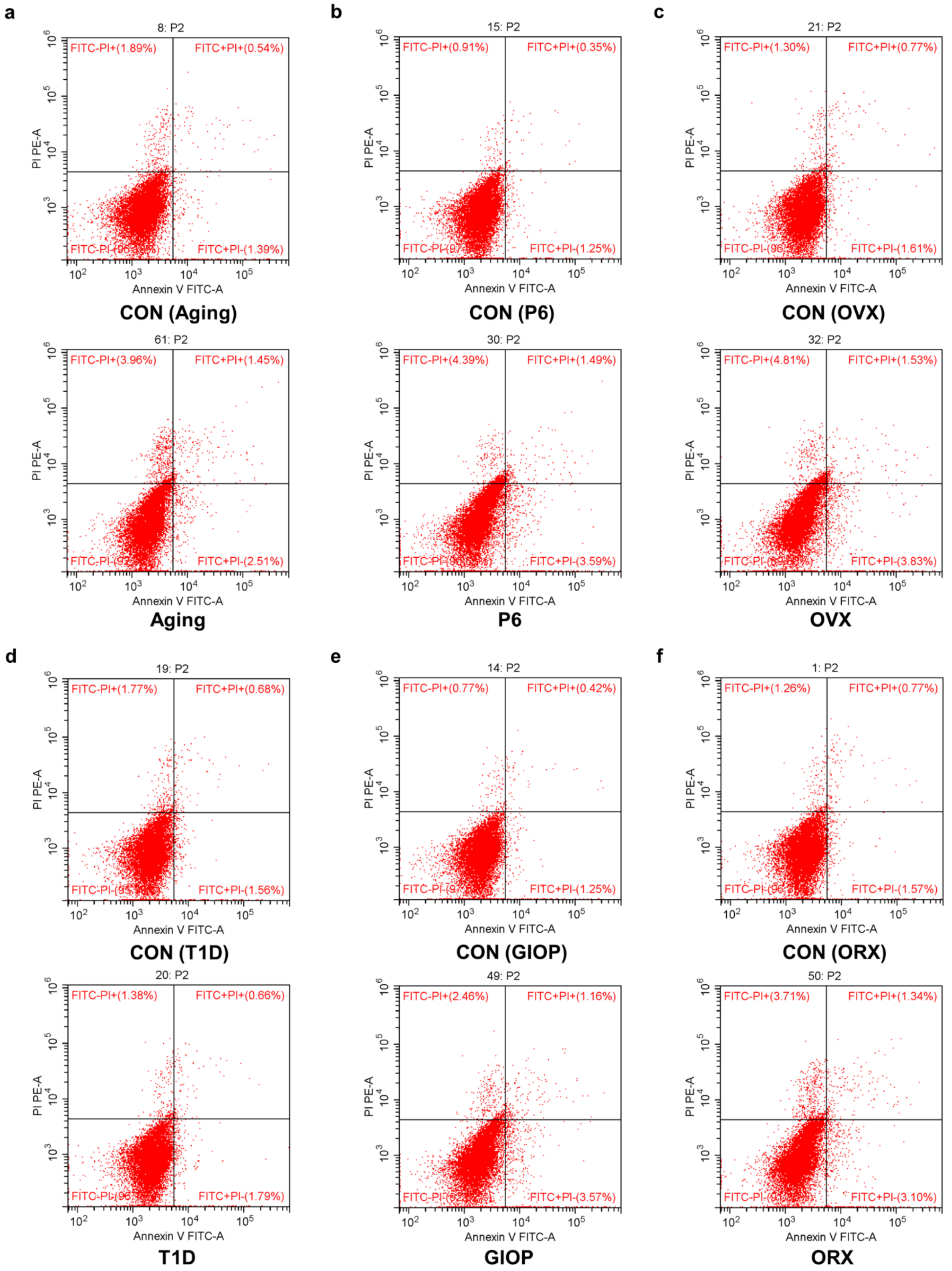

Supplementary Figure S8. Apoptosis of bone marrow stromal cells (BMSCs). (**a-f**) Representative flow cytometric images of BMSCs demonstrating apoptosis evaluated by FITC-conjugated Annexin V and

propidium iodide (PI) double staining. Mice were modeled 4 weeks for the control groups and osteoporoses induced by natural aging (**a**), accelerated senescence (SAMP6) (**b**), ovariectomy (OVX) (**c**), type 1 diabetes (T1D) (**d**), excessive glucocorticoids (GIOP) (**e**) and orchidectomy (ORX) (**f**). The percentages of early apoptotic ($FITC^+PI^-$) plus late apoptotic/necrotic ($FITC^+PI^+$) cells were expressed as apoptotic BMSC percentages.

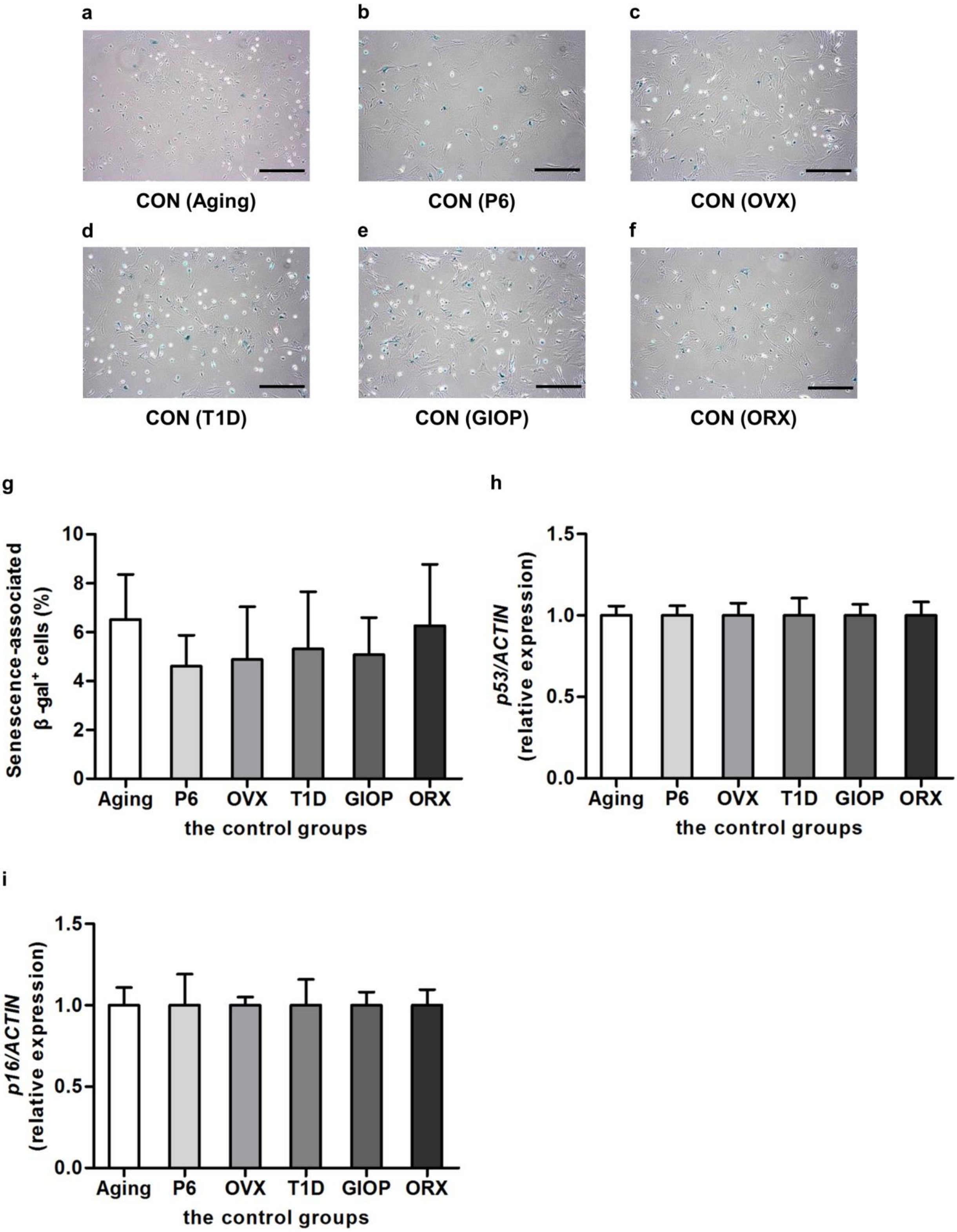

Supplementary Figure S9. Cellular senescence of bone marrow stromal cells (BMSCs) derived from the control groups. (**a-f**) Representative β-galactosidase (β-gal) staining images demonstrating cellular senescence of murine BMSCs derived from the control groups of natural aging (**a**), accelerated senescence

(SAMP6) (**b**), ovariectomy (OVX) (**c**), type 1 diabetes (T1D) (**d**), excessive glucocorticoids (GIOP) (**e**) and orchidectomy (ORX) (**f**). Mice were sacrificed after 4 weeks of modeling. 1st-passaged BMSCs were seeded at 1×10^5 cells/well in 12-well plates and were stained for β-gal. The senescent cells were positively stained (green). Bars: 200 μm. (**g**) The corresponding percentage of senescence-associated β-gal positive cells over total cells showing paralleled senescence of BMSCs in control groups of the 6 different types of osteoporoses. (**h, i**) Quantitative real-time polymerase chain reaction (qRT-PCR) analysis of the mRNA expression level of *p53* and *p16*. The corresponding values were not significantly different among the control groups. The expressions of *p53* and *p16* were normalized to that of *ACTIN*. Data represents mean ± standard deviation (SD). n=6 per group.

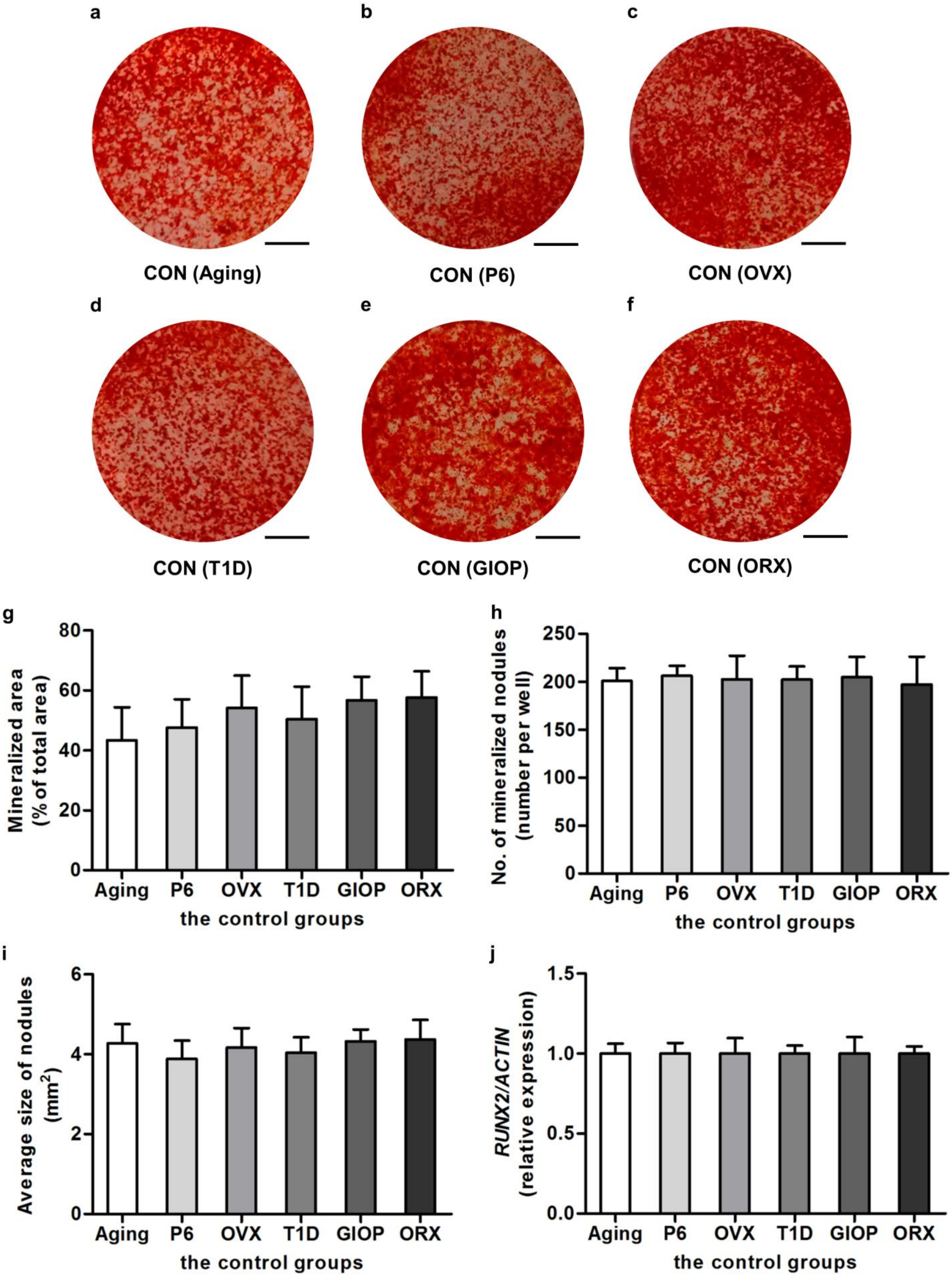

Supplementary Figure S10. Osteogenic differentiation of bone marrow stromal cells (BMSCs) derived from the control groups. (**a-f**) Representative alizarin red staining images demonstrating mineralization by

murine BMSCs derived from the control groups of natural aging (**a**), accelerated senescence (SAMP6) (**b**), ovariectomy (OVX) (**c**), type 1 diabetes (T1D) (**d**), excessive glucocorticoids (GIOP) (**e**) and orchidectomy (ORX) (**f**). Mice were sacrificed after 4 weeks of modeling. 1st-passaged BMSCs were seeded at 2×10^5 cells/well in 12-well plates and underwent osteogenic induction for 14 days. Bars: 5 mm. (**g-i**) The corresponding parameters of mineralized area over total area (**g**), number of mineralized nodules per well (**h**) and average size of nodules (**i**) showing comparable mineralization of BMSCs in control groups of the 6 different types of osteoporoses. (**j**) After 14-day osteogenic induction, quantitative real-time polymerase chain reaction (qRT-PCR) analysis of the mRNA expression level of osteogenic marker gene *Runt-related transcription factor 2 (RUNX2)*. The corresponding values were not significantly different among the control groups. The expression of *RUNX2* was normalized to that of *ACTIN*. Data represents mean ± standard deviation (SD). n=6 per group.

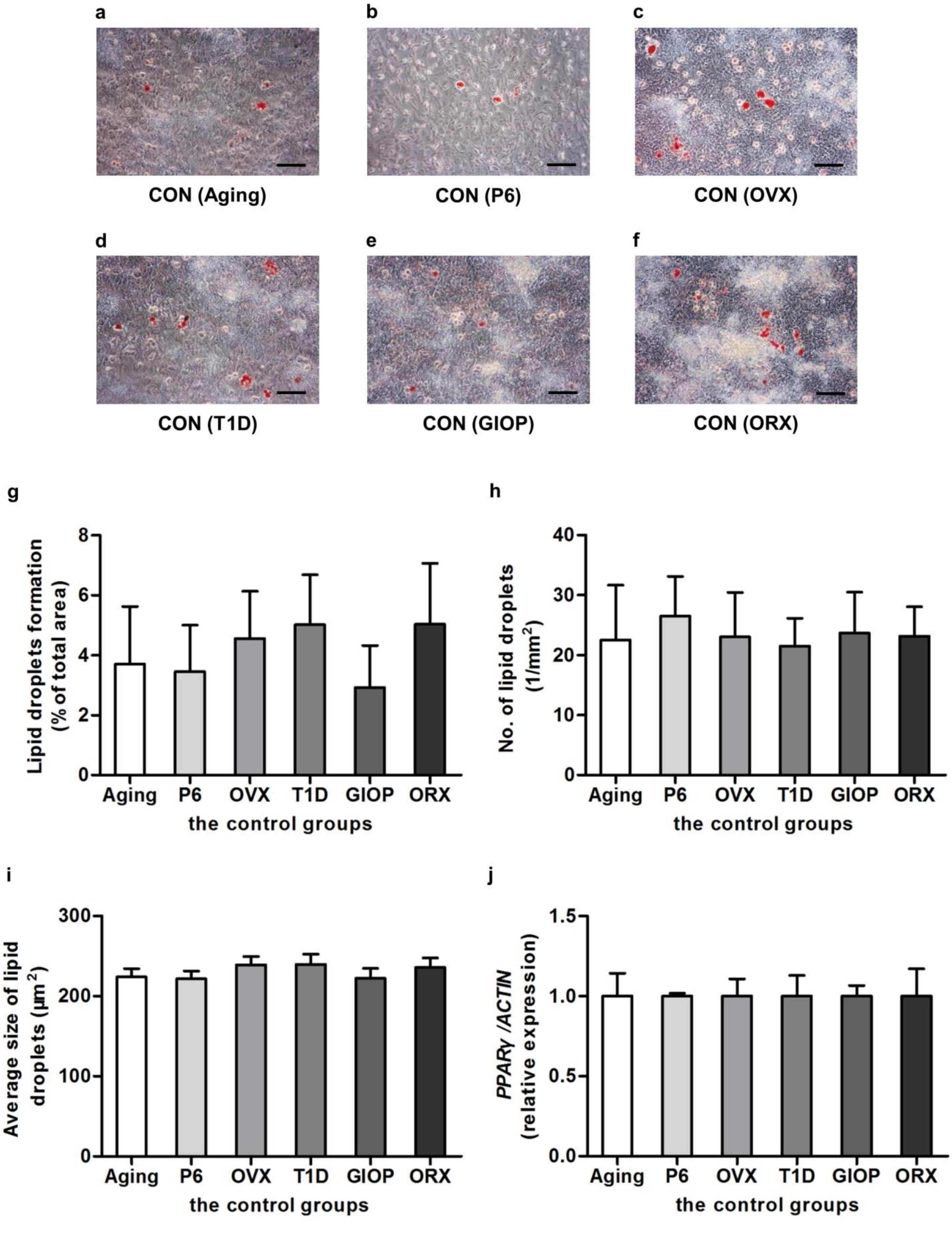

Supplementary Figure S11. Adipogenic differentiation of bone marrow stromal cells (BMSCs) derived from the control groups. (**a-f**) Representative oil red O staining images demonstrating formation of lipid droplets by murine BMSCs derived from the control groups of natural aging (**a**), accelerated senescence

(SAMP6) (**b**), ovariectomy (OVX) (**c**), type 1 diabetes (T1D) (**d**), excessive glucocorticoids (GIOP) (**e**) and orchidectomy (ORX) (**f**). Mice were sacrificed after 4 weeks of modeling. 1st-passaged BMSCs were seeded at 2×10^5 cells/well in 12-well plates and underwent adipogenic induction for 14 days. Bars: 100 μm. (**g-i**) The corresponding parameters of lipid droplets formation over total area (**g**), number of lipid droplets per square millimeter (**h**) and average size of lipid droplets (**i**) showing paralleled adipogenic differentiation of BMSCs in control groups of the 6 different types of osteoporoses. (**j**) After 14-day adipogenic induction, quantitative real-time polymerase chain reaction (qRT-PCR) analysis of the mRNA expression level of adipogenic marker gene *Peroxisome proliferator activated receptor gamma (PPARγ)*. The corresponding values were not significantly different among the control groups. The expression of *PPARγ* was normalized to that of *ACTIN*. Data represents mean ± standard deviation (SD). n=6 per group.

Supplementary Table S1. Primer sequences in the present study.

Gene	Primer sequences
ACTIN	Forward: 5’-CATCCGTAAAGACCTCTATGCCAAC-3’
	Reverse: 5’-ATGGAGCCACCGATCCACA-3’
p53	Forward: 5’-ATGGTCAAGAAAGTGGGGCCT-3’
	Reverse: 5’-TGAGTGGATCCTGGGGATTGT-3’
p16	Forward: 5’-CTCACCTCGCTTGTCACAGT-3’
	Reverse: 5’-TCGCACGAACTTCACCAAGA-3’
RUNX2	Forward: 5’-AAATGCCTCCGCTGTTATGAA-3’
	Reverse: 5’-AAGGTGAAACTCTTGCCTCGTC-3’
PPARγ	Forward: 5’-TGTGAGACCAACAGCCTGAC-3’
	Reverse: 5’-AAGTTGGTGGGCCAGAATGG-3’
